# Loss of tet methyl cytosine dioxygenase 3 (TET3) enhances cardiac fibrosis via modulating the DNA damage repair response

**DOI:** 10.1186/s13148-024-01719-6

**Published:** 2024-08-27

**Authors:** Sandip Kumar Rath, Gunsmaa Nyamsuren, Björn Tampe, David Sung-wen Yu, Melanie S. Hulshoff, Denise Schlösser, Sabine Maamari, Michael Zeisberg, Elisabeth M. Zeisberg

**Affiliations:** 1 Department of Cardiology and Pneumology, University Medical Center Göttingen, Robert-Koch-Str. 40, 37075 Göttingen, Germany; 2 Department of Nephrology and Rheumatology, University Medical Center Göttingen, Robert-Koch-Str. 40, 37075 Göttingen, Germany; 3 DZHK (German Center for Cardiovascular Research, Partner Site Lower Saxony, Göttingen, Germany; 4 Department of Radiation Oncology, Winship Cancer Institute, Emory University School of Medicine, Atlanta, GA 30322 USA

**Keywords:** TET3, Homologous recombination, Non-homologous end joining, TGF-ß, Fibrosis

## Abstract

**Background:**

Cardiac fibrosis is the hallmark of all forms of chronic heart disease. Activation and proliferation of cardiac fibroblasts are the prime mediators of cardiac fibrosis. Existing studies show that ROS and inflammatory cytokines produced during fibrosis not only signal proliferative stimuli but also contribute to DNA damage. Therefore, as a prerequisite to maintain sustained proliferation in fibroblasts, activation of distinct DNA repair mechanism is essential.

**Result:**

In this study, we report that TET3, a DNA demethylating enzyme, which has been shown to be reduced in cardiac fibrosis and to exert antifibrotic effects does so not only through its demethylating activity but also through maintaining genomic integrity by facilitating error-free homologous recombination (HR) repair of DNA damage. Using both in vitro and in vivo models of cardiac fibrosis as well as data from human heart tissue, we demonstrate that the loss of TET3 in cardiac fibroblasts leads to spontaneous DNA damage and in the presence of TGF-β to a shift from HR to the fast but more error-prone non-homologous end joining repair pathway. This shift contributes to increased fibroblast proliferation in a fibrotic environment. In vitro experiments showed TET3’s recruitment to H2O2-induced DNA double-strand breaks (DSBs) in mouse cardiac fibroblasts, promoting HR repair. Overexpressing TET3 counteracted TGF-β-induced fibroblast proliferation and restored HR repair efficiency. Extending these findings to human cardiac fibrosis, we confirmed TET3 expression loss in fibrotic hearts and identified a negative correlation between TET3 levels, fibrosis markers, and DNA repair pathway alteration.

**Conclusion:**

Collectively, our findings demonstrate TET3’s pivotal role in modulating DDR and fibroblast proliferation in cardiac fibrosis and further highlight TET3 as a potential therapeutic target.

**Graphical abstract:**

**Figure:**
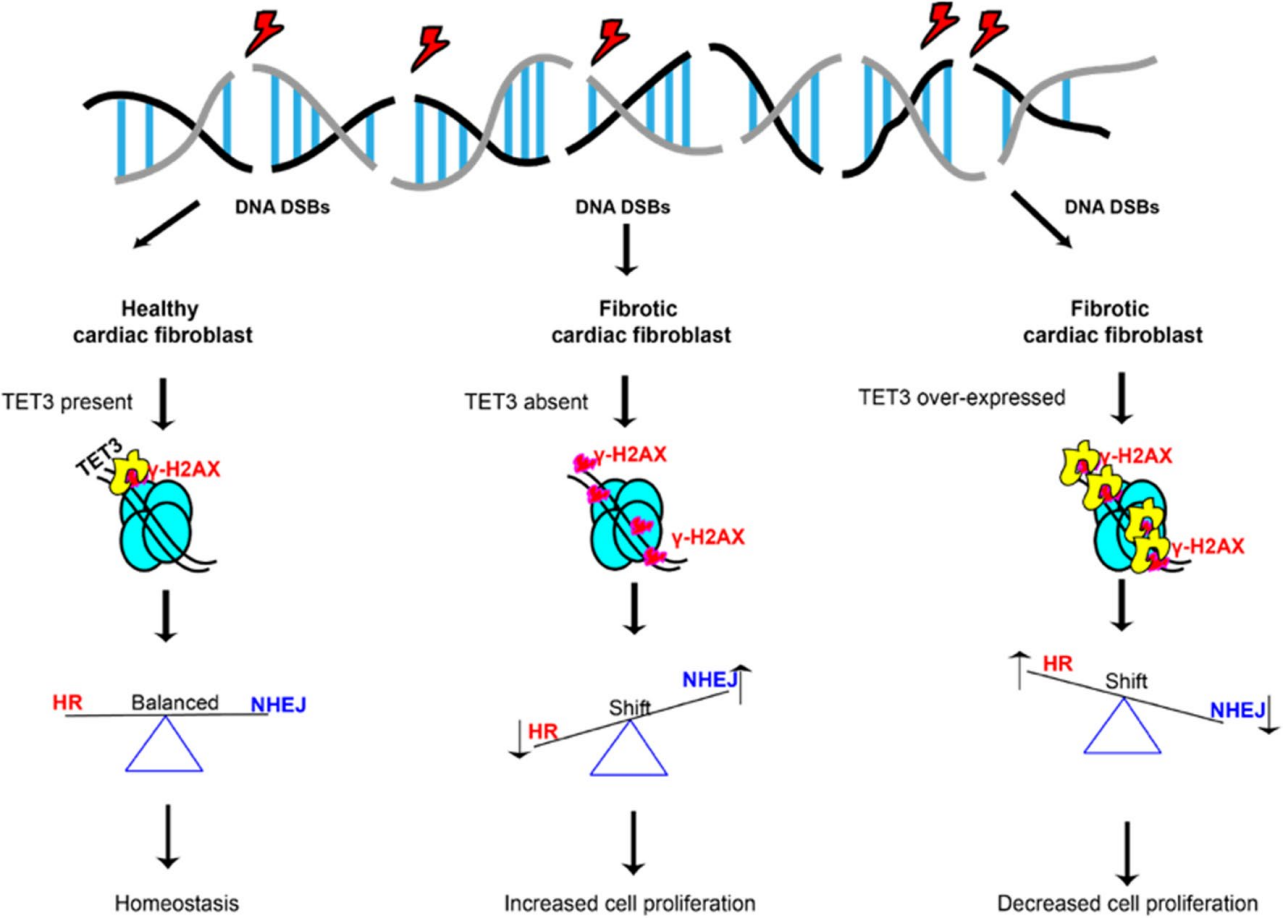
Schematic representation illustrating the role of TET3 in modulating the DDR response in healthy and fibrotic fibroblasts.

**Supplementary Information:**

The online version contains supplementary material available at 10.1186/s13148-024-01719-6.

## Background

Cardiovascular disease (CVD) is the leading cause of mortality worldwide [1, 2]. Cardiac fibrosis is a hallmark feature of most forms of CVD [2]. While cardiac fibrosis represents a normal response to myocardial injury, myo-fibroblastic proliferation and excessive accumulation of extracellular matrix (ECM) proteins can lead to increased myocardial stiffness, impaired systolic and diastolic function, and, eventually, heart failure and death [2–4]. Therefore, understanding the underlying mechanisms that drives progression of cardiac fibrosis need to be investigated, to develop effective therapeutic intervention. Fibroblasts are a non-myocytic population of heart cells that play a central role in fibrogenesis [5]. The fibrotic microenvironment is highly dynamic, with both cellular and non-cellular components affecting fibrosis. For instance, chronic accumulation of reactive oxygen species (ROS) can aggravate fibrosis [6, 7] by inducing oxidative stress, cytokine production, and DNA damage [8–11]. ROS-induced oxidative stress can cause both DNA single-strand breaks (SSBs) and double-strand breaks (DSBs) [12, 13]. Impact of DSBs is poorly studied in cardiac fibrosis and heart failure [14]. DSBs are highly deleterious to the cell and, failure to engage repair response, lead to premature transcription, replication stress, and/or apoptosis together with an increased risk of genomic instability [15]. To protect genomic integrity, the cells have a complicated and intricate network of signaling pathways to detect and repair DSBs, collectively known as DNA repair and response (DDR) pathways [16]. DSBs are primarily repaired by the two main DDR pathways; one is error-prone non-homologous end joining (NHEJ) pathway and the second is error-free homologous recombination (HR) pathway [17]. NHEJ is a fast, cell-cycle-independent DDR mechanism that restores DNA damage by ligating broken DNA ends, using little or no sequence homology [18], while HR is a slow, cell-cycle-dependent DDR mechanism that relies on DNA end resection to restore DNA integrity [15].

Emerging studies have highlighted the role of DNA damage as an independent risk factor for the development of cardiovascular disease. As discussed, proliferation of fibroblasts is essential for progression of fibrosis. To our knowledge, however, the molecular mechanism underpinning the proliferation of cardiac fibroblasts despite the presence of DNA DSBs is largely unknown. Notably, the fibrotic progression is also associated with changes in epigenetic marks [19, 20]. In line with these observations, our group has previously demonstrated a hydroxymethylation-dependent role of TET3 (a DNA demethylase) in ameliorating organ fibrosis [21, 22].

Proteins belonging to the TET family are increasingly associated with DNA repair, and ongoing studies have highlighted their roles as emerging new gatekeepers of genomic integrity [23]. A study in glial cells showed that loss of TET1 results in the activation of G2M arrest and, even in the absence of genotoxic stress, promotes an endogenous increase in DNA DSBs [24]. Similarly, loss of TET2 has been found to cause defects in chromosome segregation and a decrease in BRCA2 (involved in HR repair) mRNA expression [25]. Additionally, a recent report demonstrated that ATR-mediated stabilization of TET3 is involved in DNA repair [26]; however, the study shed no light on whether TET3 is recruited directly to DNA damage sites or its involvement in the choice of DNA repair. In line with these observations, we hypothesized that TET3 may be associated with fine tuning the proliferation of fibroblasts via regulating the DDR response. Herein, we report that both mouse and human hearts undergoing chronic fibrosis exhibit decreased TET3 expression. Using mouse cardiac fibroblasts (mCFs) as an in vitro model, we demonstrated that TET3 knockdown results in decreased HR-mediated DNA efficacy and a change in chromatin architecture. Interestingly, mimicking a fibrotic microenvironment upon TGF-β treatment in vitro in mCFs, we demonstrated that downregulation of TET3 in combination with TGF-β treatment is accompanied by a switch towards NHEJ-mediated DNA repair and thus facilitates the proliferation of cardiac fibroblasts. Finally, upon overexpression of TET3 in a pro-fibrotic niche, we demonstrated a shift in DDR balance from NHEJ- to HR-mediated DNA repair, likely resulting in decreased cell proliferation. Cumulatively, our findings provide new insights into how the shift in balance of the DDR pathways due to the loss of TET3 partially affects the proliferation of fibrotic fibroblasts during cardiac fibrosis. Thus, the present findings open new avenues for targeting the balance in DDR pathways for therapeutic intervention during cardiac fibrosis.

## Materials and methods

### Human myocardial tissue sections

All patient samples are collected from the Department of Cardiology, UMG Gottingen, in accordance with ethical rules and regulations of the Institutional Review Board of the University of Göttingen and the responsible government authority of Lower Saxony (Germany).

### Animal welfare and ethics statement

All experimental animal studies were conducted in accordance with the guidelines of the experimental protocols and ethical rules approved by the Institutional Review Board of the University of Göttingen and the responsible government authority of Lower Saxony (Germany). The animal protocols used in these experiments conformed to the guidelines in Directive 2010/63/EU of the European Parliament on the protection of animals.

### Fibrosis induction using angiotensin-II osmotic minipump in mice

The angiotensin-II model was implemented as described. Briefly, 14- to 16-week-old C57/BL/6N mice with body weights between 25 and 30 g were used for angiotensin-II (Ang-II) pump implantation experiments. Ang-II (1.5 mg/kg per day), or PBS as the control, was administered to the animals, using an osmotic minipump (ALZET Model 1002), for 4 weeks.

### Cell culture

Mouse primary cardiac fibroblast (mCFs) was obtained from Cell-Science. The cells were cultured using 1.5 g Glucose Dulbecco’s Modified Eagle Medium (DMEM) supplemented with 10% fetal bovine serum, sodium pyruvate (1 mM), nonessential amino acids (0.1 mM), penicillin (100 units/ml), and streptomycin (0.1 mg/ml) and maintained at 37 °C in 5% CO_2._

### Neocarzinostatin (NCS) and transforming growth factor beta (TGFβ) treatment

The mCFs were treated with either 100 ng/mL of neocarzinostatin (NCS) or 15 ng/mL of transforming growth factor beta (TGF-β) in all subsequent cell culture experiments.

### Generation of clustered regularly interspaced short palindromic repeats/CRISPR-associated protein 9 (CRISPR/Cas9) tet methylcytosine dioxygenase 3 (*TET3*) knockdown constructs

The clustered regularly interspaced short palindromic repeats/CRISPR-associated protein 9 (CRISPR/Cas9) backbone was used to generate tet methylcytosine dioxygenase 3 (*TET3*) gene knockdown constructs in the MCF. Guided RNAs targeting exon 10 and exon 11 of the *TET3* gene were designed, and off-target binding effects were minimized on the basis of scores obtained on the online tool Blueheronbio (OriGene, Herford, Germany). The single-guide RNA (sgRNA) sequences were inserted into the pLenti-Cas9-Guide plasmid (OriGene GE100010, Herford, Germany) with BamHI and BsmBI restriction sites to generate p-Lenti-Cas-sgRNA mTET3 constructs and confirmed by DNA sequencing. The deletion of the flanked exon was predicted to lead to a frameshift mutation, eventually resulting in the generation of a premature spliced transcript, leading to decreased protein expression.

### In vitro transfection

The night before transfection, 5 × 10^4^ cells per well were seeded in a 6-well culture plate in an antibiotic-free DMEM (Gibco, Carlsbad, USA) well supplemented with 10% heat-inactivated fetal bovine serum (FBS, Sigma-Aldrich, St. Louis, USA). For the knockdown experiments, 2.5 µg of pLenti-cas9 *TET3* plasmid DNA was transfected; for overexpression, 2.5 µg of mouse *TET3* plasmid DNA was transfected using Lipofectamine 2000 reagent (Invitrogen, Carlsbad, USA). A total of 4 h after transfection, the medium in each well was replaced by a fresh antibiotic-free medium and allowed to incubate for the next 48 h.

### Histology and immunohistochemistry

All histology and immunohistochemistry experiments were performed as described in our previous publications [20, 22].

### Single-cell, neutral gel electrophoresis

Neutral comet assay was performed on the whole mouse hearts and in vitro in mouse cardiac fibroblasts (MCF). Briefly, isolated cells from the mouse hearts or MCF were mixed with 1% low-melting agarose gel. The resulting solution was then poured on a chilled precoated agarose glass slide. The cells were then lysed overnight at 4 °C in lysis solution. The next day, the slides were run in the freshly prepared neutral running buffer for 30 min at 12 V. Post-electrophoresis, SYBR Safe was added to visualize the comet tails using a fluorescent microscope.

### Amplex Red assay

The H_2_O_2_ concentration in the mouse hearts was measured using the Amplex Ultrared dye according to the manufacturer’s instructions. In brief, the mouse hearts were minced into small pieces (25 mg) and incubated with Amplex Red at a concentration of 100 μmol/L and horseradish peroxidase at a concentration of 1 U/mL for 60 min in the dark. The supernatant was collected immediately after incubation and transferred to a black-coated 96-well plate, and fluorescence was measured at 560 nm.

### Glutathione/glutathione disulfide (GSH/GSSG) activity assays

Both activity assays were performed in the mouse hearts as per the manufacturer’s instructions (ab156913, ab138881).

### Immunofluorescence

A total of 10 000 cells per chamber were seeded in the 8-well chambered slides. Before fixing, the slides were washed twice with 1X PBS. Fixing was performed using 4% paraformaldehyde (PFA) for 15 min at room temperature. Post-fixation cells were permeabilized with 0.1% phosphate-buffered saline with Tween 20 (PBST) (1XPBS + Triton X 100) for 7 min; they were then washed twice in 1X PBS. The cells were blocked with 5% bovine serum albumin (BSA) in PBST for 1 h at room temperature. Post-blocking, the cells were incubated with respective primary antibodies dissolved in 1% BSA in PBST overnight at 4 °C. The next day, the cells were washed thrice with 1X PBS and thereafter incubated with secondary antibody dissolved in 1% BSA in PBST for 1 h. The cells were rewashed thrice with 1X PBS and mounted with 4′,6-diamidino-2-phenylindole (DAPI) to be visualized under the microscope.

### Confocal image analysis

All images were photographed using an LSM780 confocal microscope. Triple-stained images were taken using settings in the frame with either Alexa green 488, Alexa red 568, or Alexa infrared 647 lasers. All the images represented were processed using ZEN blue software (ZEISS, Oberkochen, Germany), keeping the parameters constant. The nuclei of all the represented images were visualized with the DAPI channel.

### BrdU DNA end resection assay

Briefly, control, TET3 knockdown, and TET3 rescued mCFs were incubated with 20 mM of BrdU for 24 h. Cells were fixed with 90% ethanol in 1X PBS for 20 min. After fixation, cells were washed thrice with 0.1% PBST and then subjected to non-denaturing conditions. Cells were subsequently blocked with 10% FBS in 1X PBS for 1 h at room temperature and thereafter washed thrice with 1X PBS. Subsequently, anti-BrdU secondary antibody from the BrdU kit was added and absorbance was measured in an ELISA plate reader at 370 nM.

### RNA isolation and RT-PCR

RNA isolation, synthesis of complimentary cDNA, and real-time PCR were performed as previously described in our publications [20, 21].

### Western blot

Western blot was performed as previously described in our publications [20, 21].

### Global 5-hydroxymethylcytosine (5-hmC) detection assay

Total DNA was isolated from mouse cardiac fibroblasts using the DNeasy Blood and Tissue Kit (Qiagen) following the manufacturer’s instructions. The purified DNA was then used to detect the global content of 5-hmC (MethylFlash Hydroxymethylated DNA Quantification Kit, Epigentek (Catalog Number-P-1032-96)). The chemiluminescent signal was collected using the plate reader (PerkinElmer Inc.). Each experimental plate contained duplicate measurements of the samples, and positive DNA control samples, provided by the manufacturer, were used as inter-run calibration samples. The sensitivity of the assay according to the manufacturer is 0.02%.

### Flow cytometry

Briefly, the cells were washed with PBS twice before detachment with trypsin (1:3 dilution, incubation for 2 to 4 min at 37 °C). An equal amount of culture media was added to stop the reaction. The cell solution was centrifuged for 10 min with 1.200 RPM, and the supernatant was discarded. Afterward, the cells were washed twice with ice-cold PBS; this involved carefully suspending them in 1 ml of PBS before centrifuging them with 1.200 RPM for 10 min. In a final step, the cells were suspended in 500 µl of ice-cold PBS. A total of 5 ml of 70% ethanol was added dropwise with constant vortexing. The cells were in single suspension after this procedure; they were frozen at -80 °C overnight for at least 1 h. In the next step, the suspension was centrifuged again for 10 min with 4.600 RPM at 4 °C. The supernatant was discarded, and the precipitate was resuspended in 500 µl of ice-cold PBS. The solution was transferred into Eppendorf tubes. Afterward, 500 µl of propidium iodide and 2 µl of ribonuclease A (RNase A) were added and mixed conscientiously. The suspension was incubated for 30 min at 37 °C. Within 1 h, the measurement was taken with BD Accuri TM C6 (BD Biosciences, San Jose, California, USA).

### Proximity ligation assay (PLA)

The cells were seeded at a density of 10^4^ cells per well in an 8-chambered slide. The cells were fixed and permeabilized as described before in immunofluorescent studies. After permeabilization, the cells were incubated with blocking buffer provided in the mouse/rabbit red starter Duolink kit (Olink, Uppsala, Sweden) for 2 h at 37 °C in a humidified chamber. The primary antibodies were then conjugated with the probes provided within the kit and incubated for 1 h at room temperature at 37 °C in a humidified chamber. They were then washed 3 times in Buffer A (provided in the kit). The cells were then combined with amplification buffer and enzymes as per the manufacturer’s protocol and incubated for 90 min at 37 °C in a darkened humidified chamber. Finally, the cells were washed with 1 × Buffer B (supplied with the kit) for 10 min; this was followed by a 1-min wash with 0.01X Buffer B. Finally, the cells were mounted using the DAPI conjugated mountant supplied with the kit. The red blobs indicated the proximity between 2 cellular-bound antibodies. For graph representation, the number of detected red blobs was counted per well, with 100 cells each, from 3 independent experiments.

### Non-homologous end joining (NHEJ) and homologous recombination (HR) reporter plasmids

The mCFs were stably transfected with 2.5 µg of circular pCLN-DSB or pDR-GFP (Addgene, Cambridge, USA) (Pierce et al., 1999; Seluanov et al., 2004). Resistant colonies were selected with 5 µg/mL of puromycin (Thermo Fisher Scientific, Waltham, USA). Transfection with I-SceI (pCBASceI, Addgene, Cambridge, USA) introduced a double-strand break (DSB) at genomic I-SceI sites of the reporter plasmid, which helped to restore the green fluorescent protein/enhanced green fluorescent protein (GFP/EGFP) signal, visualizing non-homologous end joining (NHEJ)/homologous recombination (HR) repair events.

### Cell counting assay

Briefly, the cells were counted using the trypan blue assay. The rate of proliferation was calculated as *R*^*p*^ = ln(N(*t*)/N (0))/*t,* where *N*(*t*) = the number of cells at time *t*, *N* (*0*) = the number of cells at time 0, and *t* = time (in days).

### MTT cell proliferation assay

Briefly, 1000 cells were plated in a 96-well chambered plate and 20 μl of 5 mg/ml MTT was added to each well. One set of wells with MTT but without cells was taken as negative control. After addition of MTT, cells were left to incubate at 37 °C for 4 h. After incubation, media was removed and 100 μl of DMSO was added. Briefly, 10 min within incubation, cells were measured for proliferation at 495 nm using an ELISA plate reader.

### MNase digestion assay

Briefly, mCFs were resuspended in ice-cold lysis buffer containing 10 mM Tris–HCl (pH 7.5), 10 mM NaCl, 3 mM MgCl2, 0.1% IGEPAL CA-630, and 1 mM DTT, supplemented with a protease inhibitor cocktail to isolated nuclei. Subsequently, the lysate was centrifuged to separate nuclear pellets from the supernatant. The nuclear pellets were resuspended in ice-cold nuclear resuspension buffer comprising 20 mM Tris–HCl (pH 7.5), 150 mM NaCl, 3 mM MgCl2, and 0.1% Triton X-100, followed by centrifugation to re-pellet the nuclei. For MNase digestion, the purified nuclei were resuspended in MNase digestion buffer consisting of 15 mM Tris–HCl (pH 7.5), 15 mM NaCl, 60 mM KCl, 0.1 mM EGTA, and 0.5 mM spermidine. The appropriate amount of MNase enzyme was then added to initiate chromatin digestion, allowing MNase to preferentially cleave accessible regions of chromatin. After the digestion reaction, the reaction was terminated by adding 1 M EDTA to the mixture. DNA fragments generated from this digestion represented regions of chromatin accessibility. The DNA was subsequently purified using phenol–chloroform extraction followed by ethanol precipitation and analyzed using bioanalyzer.

### Analysis of publicly available microarray datasets

Datasets provided publicly were analyzed according to general recommendations, using Transcriptome Analysis Console software (Thermo Scientific, Waltham, Massachusetts, USA). Human transcriptome array data were shown as log2 median-centered intensities extracted from database accession numbers **GSE57345**.

### Statistical analysis

Statistical analysis was performed using Graph Pad Prism 8 software. For comparing between two groups unpaired two-tailed Student’s t test was performed. All experiments were performed in biological triplicates. For human heart samples, data were obtained from 5 donor patients (5 non-fibrotic vs 5-fibrotic donor human heart samples). For comparison of more than two groups, one-way ANNOVA analysis was performed. Statistical significances are represented in the graphs as *p ≤ 0.05, **p ≤ 0.01, ***p ≤ 0.001.

## Results

### Mouse fibrotic hearts have increased ROS, oxidative stress, and DNA damage but are still able to induce the proliferation of fibroblasts

**Fig. 1:**
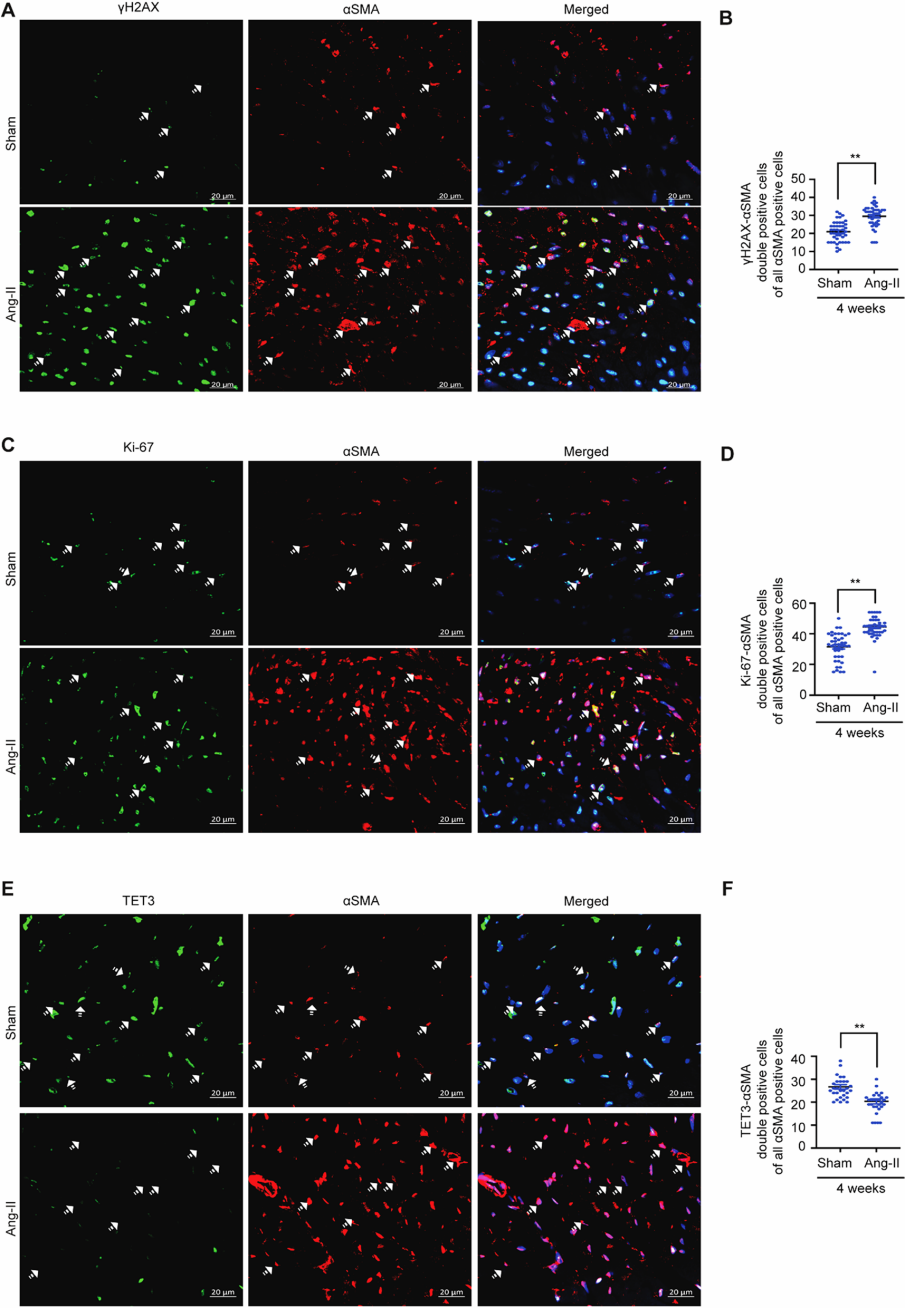
Mouse fibrotic fibroblasts proliferate despite increased DNA damage. **A–B** Confocal representative images and respective graphs show double staining of γH2AX (a marker of DNA double-strand breaks) and αSMA (a marker of fibroblast) in 4 weeks of sham and angiotensin-II-treated mouse hearts. **C–D** Confocal representative images and respective graphs show double staining of ki67 and α- SMA in 4 weeks of sham and angiotensin-II-treated mouse hearts. **E–F** Confocal representative images and respective graphs showing double staining of TET3 and αSMA in 4 weeks of sham and angiotensin-II-treated mouse hearts. Scale bars represent 20 μm. In total, each dot in the graph represents the fibrotic area analyzed from 5 independent fields from three independent samples (Sham vs Angiotensin-II). Summarized quantitative findings are shown as mean ± SEM from three shams, and 3 angiotensin-II-treated mouse hearts. Statistical significance was calculated using two-tailed Student’s t test, and P values correspond to *p ≤ 0.05, **p ≤ 0.01, ***P ≤ 0.01

The fibrotic microenvironment is highly dynamic, and studies have shown that chronic accumulation of ROS causes oxidative stress in fibrotic hearts and leads to cytokine production and DNA damage [6, 27, 28]. In line with these observations, we investigated the production of ROS (H₂O₂) and the induction of oxidative stress in a murine model of cardiac fibrosis treated with angiotensin-II. Using Masson’s trichrome staining (MTS), we confirmed fibrotic induction (Fig. s1A–B). Next, we confirmed a 3.7-fold increase in ROS generation in the fibrotic hearts as compared to healthy mouse hearts (Fig. s1C). Increased ROS is known to trigger induction of oxidative stress and DNA damage. Thus, we proceeded to investigate the ratio of reduced/oxidized glutathione (GSH/GSSG), a marker for confirming oxidative stress in fibrotic hearts [29]. Indeed, our results demonstrated a 1.67-fold decrease in the GSH/GSSG ratio in the fibrotic mouse hearts (Fig. s1D), which is in sync with previous studies [30]. Increased ROS and oxidative stress during cardiac fibrosis cause DNA DSBs [31, 32]. Thus, using γH2AX (a DNA DSB marker), we subsequently assessed the production of DSBs [33]. Immunohistochemical scoring showed significant production of DSBs in both myocyte and non-myocyte cell populations in the fibrotic mouse hearts as compared to healthy hearts (Fig. s1F–G). Fibroblasts comprise around 56% of the total non-myocyte cell population [34] and are prime mediators of fibrogenesis [35]. Our results demonstrated a significant increase in the percentage of γH2AX and αSMA (a fibroblast marker) double-positive cells in the fibrotic fibroblasts of the fibrotic mouse hearts as compared to the non-fibrotic fibroblasts in healthy hearts (Fig. 1A–B). Studies have shown that the presence of DSBs halts cell proliferation until the DSBs are resolved [16], but interestingly, our results demonstrated an increase in Ki67 and αSMA double-positive cells in the fibrotic fibroblasts as compared to healthy fibroblasts **(**Fig. 1C–D**)**. In sum, from our results, we confirmed that fibrotic fibroblasts proliferate despite DNA damage.

### Mouse fibrotic hearts lose TET3 expression

We have previously established that TET3 plays a protective role in organ fibrosis [20–22, 36]. Coherent with these findings, our immunohistochemistry scoring, and mRNA expression analysis demonstrated that TET3 is significantly downregulated in fibrotic mouse hearts as compared to control mouse hearts (Fig. s1E). Additionally, immunohistochemistry scoring reveals a reduction of TET3, mostly in non-cardiomyocytes (i.e., fibroblasts and endothelial cells) (Fig. s1H–I). Furthermore, by performing a co-immunofluorescent staining of TET3 with αSMA, we confirmed a 1.3-fold decrease in the total percentage of TET3 and αSMA double-positive cells in the fibrotic fibroblasts of the fibrotic mouse hearts (Fig. 1E–F). Accumulation of ROS can alter the catalytic activity of TET proteins [37, 38]. We also investigated whether the catalytic activity of TET proteins in fibrotic mouse hearts is affected. Our findings confirmed that there is a reduction in overall TET activity under these conditions (Fig. s1J).

### *TET3 is recruited to the DNA DSBs *in vitro* in mCFs when challenged with H₂O₂ and neocarzinostatin (NCS)*

**Fig. 2:**
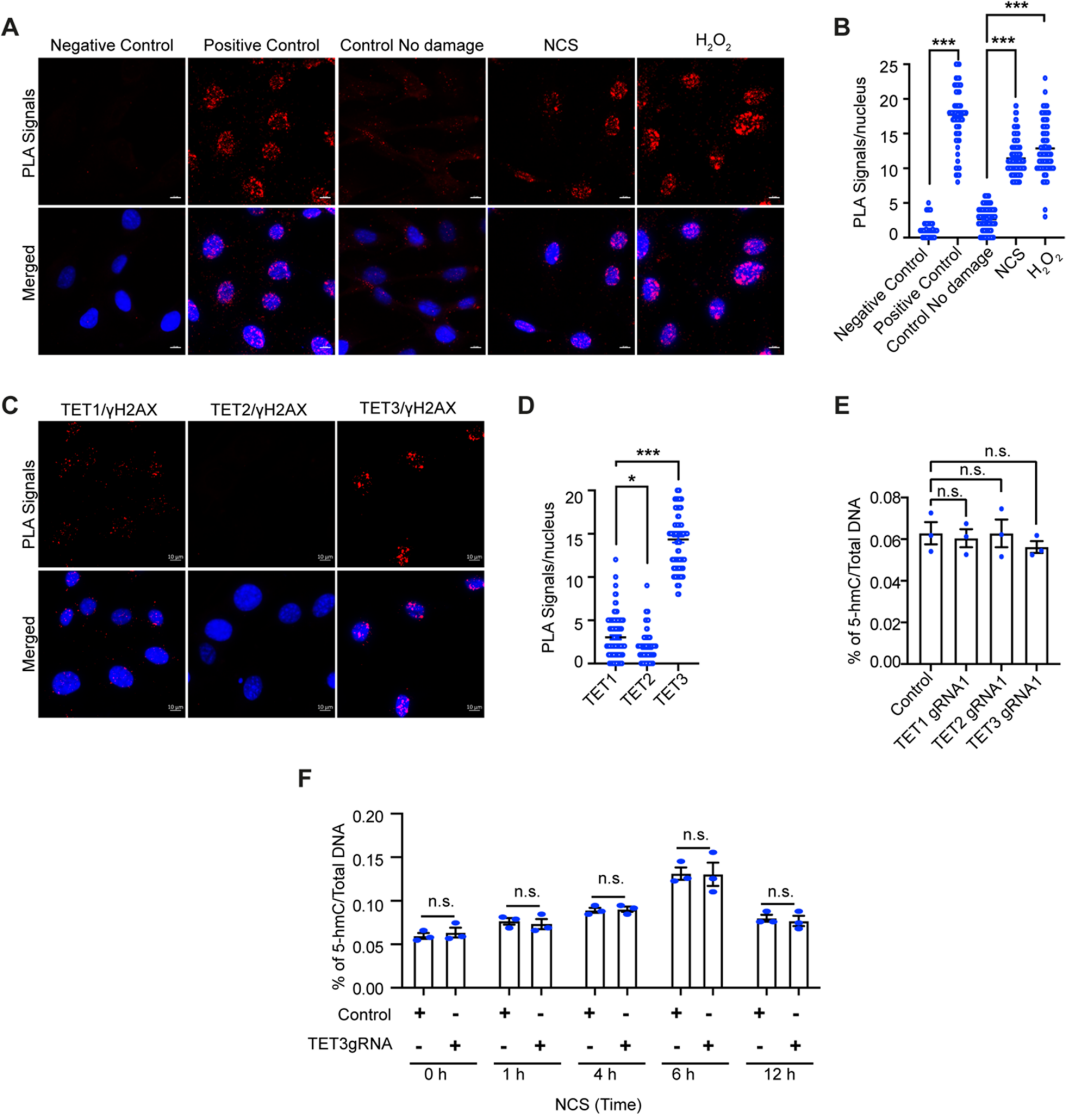
TET3 recruits to DNA DSBs in vitro in MCF independent of its 5-hmC activity. **A–B** Representative images and analysis of proximity ligation assay confirming the co-localization of **TET3** at γH2AX foci upon induction of DNA damage. Negative control represents no antibody treatment, and positive control represent 53BP1 and γH2AX. **C–D** Representative images and analysis of proximity ligation assay confirming the co-localization of TET1, TET2, or TET3 at γH2AX foci upon NCS-induced DNA damage. **E** Effect on global change in 5-hmC levels upon knockdown of TET1, TET2 and TET3. **F** Effect on global change in 5-hmC levels upon NCS-induced DNA damage in TET1 or TET3 knockdown cells. For counting, 150 cells were considered from 3 independent experiments. Statistical significance was calculated using one-way ANOVA parametric analysis, n.s. represents non-significant, and P values correspond to *p ≤ 0.05, **p ≤ 0.01, ***P ≤ 0.01. Scale bars represent 10 μm.

Recent studies have highlighted that TET3 likely plays a role in DDR [26]. In line with these observations, we proceeded to investigate the role of TET3 in DDR responses in an in vitro model of mouse cardiac fibroblasts **(smCFs)**. From our Amplex Red assay in in vivo, we confirmed that H₂O₂ is one of the critical factors contributing to DNA damage (Fig. s1C). Thus, we first examined the recruitment of TET3 to H₂O₂-induced DNA damage in vitro in smCFs at different time points. Using γH2AX and TET3 co-staining, we demonstrated that TET3 co-localizes as distinct small foci at sites of DSBs (Fig. s2A–B). Moreover, we noticed that the co-localization of TET3 at DNA lesions is at its highest an hour after H₂O₂ treatment; this peak is followed by a decline (Fig. s2B). Nevertheless, our results demonstrated that a one-time H_2_O_2_ treatment engenders continuous endogenous ROS release (Fig. s2E) and hence generation of a bimodal DNA DSBs pattern, introducing a limitation to the study of the kinetics of the recruitment of TET3 at γH2AX foci (Fig. s2B). To resolve this, we used neocarzinostatin **(NCS)** to induce DNA DSBs, as it has a short half-life and displays controlled repair kinetics (Fig. s3C–D). Our results demonstrated that treatment with NCS results in recruitment of TET3 at DSBs, which peaks after an hour of DNA damage. Notably, in the NCS-treated mCFs, the recruitment of TET3 at DNA DSBs started to decline sharply as the DNA DSBs began resolving, suggesting that TET3 follows similar recruitment kinetics to those of γH2AX. Next, by using sensitive proximity ligation assay (PLA) assay, we provided additional robust confirmation of the recruitment of TET3 at the γH2AX sites upon both NCS and H₂O₂ treatment (Fig. 2A–B). We ensured the effectiveness of the experiment by monitoring the recruitment of 53BP1 at γH2AX sites, used as a positive control (Fig. 2A–B). Similarly, to avoid false-positive results, we used no probes as a negative control (Fig. 2A–B).

### Loss of TET3 is associated with spontaneous DNA damage in mCFs

Next, to assess the role of TET3 in DDR, we generated a CRISPR/Cas9-based knockdown construct, using guide RNAs targeting exon 10 and exon 11 (Fig. s2F). Our mRNA expression data indicated that the two designed guide RNAs are equally effective in downregulating TET3 (Fig. s2G); we later confirmed this at the protein level using Western blot (Fig. s2H–I). Next, by conducting mRNA expression analysis on all TET isoforms, we ensured that knockdown of TET3 has no additional or off-target impacts on TET1 or TET2 expression (Fig. s2J). Interestingly, TET3 knockdown in mCFs using both guide RNAs results in the accumulation of endogenous DSBs (Fig. s2K–L). Notably, we observed no significant difference in the generation of DNA DSBs using both guide RNAs (Fig. sL), suggesting that the observed effect is not due to the CRISSP/Cas9 backbone. As our Western blot data showed that guideRNA2 was more effective in downregulating TET3 expression, we elected to use it for performing all future experiments. Further, using neutral comet assay, we reconfirmed the accumulation of DNA DSBs as an increase in the tail moment (head DNA% × length of tail) upon TET3 knockdown by guideRNA2 (Fig. s2M–N). We further explored whether TET3 expression levels were altered following DNA damage induction with NCS. Our results demonstrated a modest time-dependent increase in TET3 expression in mCFs (Fig. s2O).

### TET3 recruitment to the DSBs is independent of the induction of 5-Hydroxymethylcytosine (5-hmC)

All isoforms of the TET family are involved in DDR responses. One of the questions that remain unanswered, however, is whether in the absence of TET3 the two other TET isoforms can participate in DNA DSBs repair. To investigate this, we again made use of PLA assay and compared the recruitment of TET1 and TET2 at γH2AX sites upon NCS-induced DNA DSBs. Our results demonstrated that, apart from TET3, TET1 is the only isoform to be recruited at the DSBs within 1 h of NCS treatment (Fig. 2C–D). Nevertheless, the number of PLA positive dots observed for TET1 and γH2AX was statistically lower than the number observed for TET3 and γH2AX (Fig. 2D). Interestingly, our PLA results revealed negligible recruitment of TET2, as compared to TET1 and TET3, at γH2AX sites, suggesting that of all the TET isoforms, TET3 is the one that is predominantly recruited at the DNA DSBs. The TET proteins are best known for their role in DNA hydroxymethylation, but new studies highlighting a non-catalytic role of TET3 are emerging [39–41]. Therefore, it is possible that this independent catalytic activity of TET3 may play a role in the DDR response. To address this, we next assessed the change in the DNA hydroxymethylation pattern in the mCFs. Interestingly, our results showed a time-dependent increase in 5-hmC levels upon NCS-induced DNA damage (Fig. 2F), which is in sync with previous studies [42, 43]; however, no change in 5-hmC levels was observed upon TET3 knockdown in mCFs (Fig. 2E). Similar results were also obtained upon the knockdown of TET1 and TET2 proteins (Fig. 2E). Notably, we also demonstrated unchanged 5-hmC levels in TET3 knockdown mCFs challenged with NCS (Fig. 2F), suggesting that TET3 likely plays a role in the DDR response in mCFs, independent of its catalytic function.

### Knockdown of TET3 results in decreased HR but unchanged NHEJ efficacy in mCFs

**Fig. 3:**
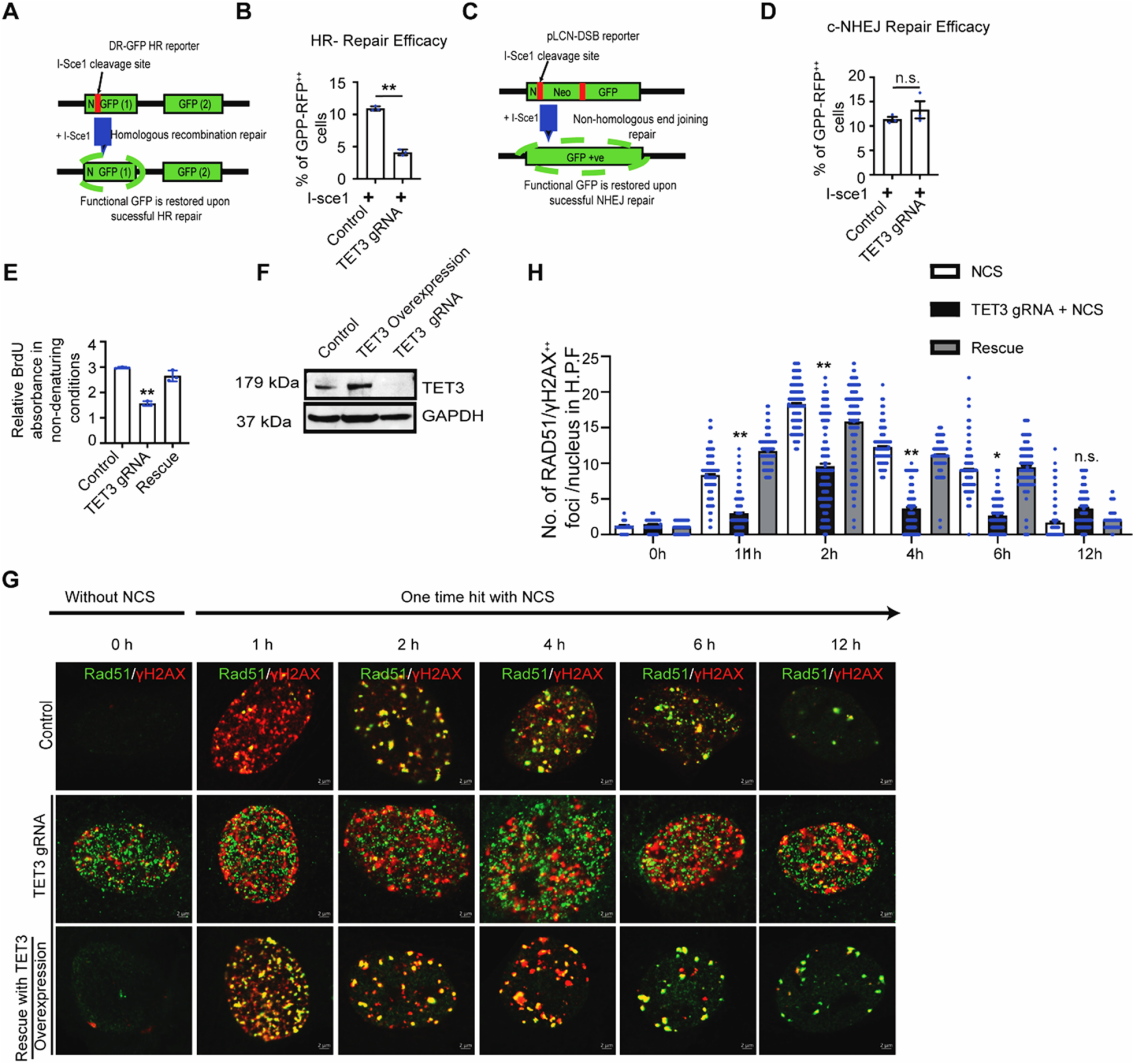
TET3 knockdown impairs HR-mediated DNA repair efficacy in vitro in mCFs. **A–B** mCFs integrated with a DR-GFP HR reporter substrate were transfected with TET3 knockdown construct and I-SceI and analyzed for change in HR efficiency by scoring % of GFP/RFP double-positive cells using flow cytometry. The associated graph represents HR efficacy in the ratio % of GFP/RFP double-positive cells. **C–D** mCFs integrated with a pLCN-DSB NHEJ reporter substrate were transfected with TET3 knockdown construct and I-SceI and analyzed for change in NHEJ efficiency by scoring % of GFP/RFP double-positive cells using flow cytometry. **E** Bar graph representing a decrease in BrdU incorporation under non-denaturing condition, a direct readout for DNA end resection in TET3 knockdown mCFs which can be rescued upon TET3 overexpression. **F** Western blot confirming TET3 knockdown and rescue in mCFs. **G** and **H** Representative confocal images and an associated graph shows rad51 (green) and γH2AX (red) co-localization (yellow) in mCFs in control, TET3 knockdown, and rescued cells (*n* = 100 cells were analyzed in each condition from 3 different experiments). For comparing between two groups two-tailed Student’s t test was performed. For comparison between more than two groups, statistical significance was calculated using one-way ANOVA parametric analysis, n.s. represents non-significant and P values correspond to *p ≤ 0.05, **p ≤ 0.01, ***P ≤ 0.01

Next, we assessed the role of TET3 in DSBs repair pathways. DSBs are predominantly repaired by either HR or NHEJ [15]. To evaluate the role of TET3 in these repair pathways, we made use of two DNA repair reporters: DR-GFP HR (to detect HR efficiency) and pLCN-DSB (to detect NHEJ efficiency) [44]. Our results revealed a significant reduction in HR repair efficiency upon knockdown of TET3, whereas NHEJ repair efficiency remained unaffected (Fig. 3A–D), suggesting that TET3 is indeed necessary for mediating HR repair response.

### Knockdown of TET3 results in decreased DNA end resection in mCFs

Diminished HR efficacy can result from either inefficient DNA end resection or improper resolution of D-loops by DNA resolvases [45–47]. Improper resolution of D-loops engenders genomic instability, increased anaphasic bridges, and micronuclei formation [47, 48]. As no such abnormal features were observed upon TET3 knockdown, we hypothesized that decreased DNA end resection can dampen HR efficacy. To investigate this hypothesis, we contemplated two strategies. First, we performed a BrdU incorporation exposure under non-denaturing conditions, which is well known to monitor direct readout for DNA end resection [49]. Our results indicated that TET3 knockdown in mCFs causes a decrease in BrdU absorbance that can be partly rescued upon re-expression of TET3 (Fig. 3E). Knockdown and rescue efficacy was confirmed in Western blot (Fig. 3F).

Second, we examined the recruitment of radiation-sensitive protein 51 (RAD51), (a core HR repair factor that facilitates the alignment and pairing of homologous DNA strands during double-strand break repair) at the DNA DSBs upon TET3 knockdown [50] Notably, our results revealed that although RAD51 expression is upregulated in TET3 knockdown cells, its recruitment to sites of DNA lesions is impaired despite an increase in DSBs (Fig. 3G-H). Even when knockdown cells are treated with NCS, this effect persists and can only be resolved upon re-expression of TET3. This suggests that the absence of TET3 does indeed affect the HR machinery via not engaging in proper loading of RAD51 at DNA DSBs, resulting in decreased DNA end resection. Interestingly, knockdown of TET3 in mCFs does not lead to decreased recruitment of Tumor Protein P53 Binding Protein 1 (53bp1) at γH2AX foci (Fig. s3A–B), suggesting that the loss of TET3 does not alter the NHEJ machinery.

Compacted chromatin limits the efficacy of HR [51]. In line with these observations, we hypothesized that impaired DNA end resection and improper loading of RAD51 to the DNA DSBs upon TET3 knockdown in mCFs can be a consequence of alteration in the chromatin architecture. To test this hypothesis, we used MNase assay, which allows to examine the chromatin accessibility in mCFs. Our results indicated that TET3-deficient mCFs have more compacted chromatin than healthy mCFs (Fig. s3C–D). Interestingly, upon rescue by TET3 overexpression, TET3 knocked down mCFs are able to relax the chromatin to a considerable extent, rendering it comparable to the chromatin in healthy mCFs; thus, supporting our hypothesis that alteration in chromatin architecture engenders diminished DNA end resection, affecting HR efficacy.

### TGF-β treatment results in decreased TET3 expression but increased proliferation in mCFs

**Fig. 4:**
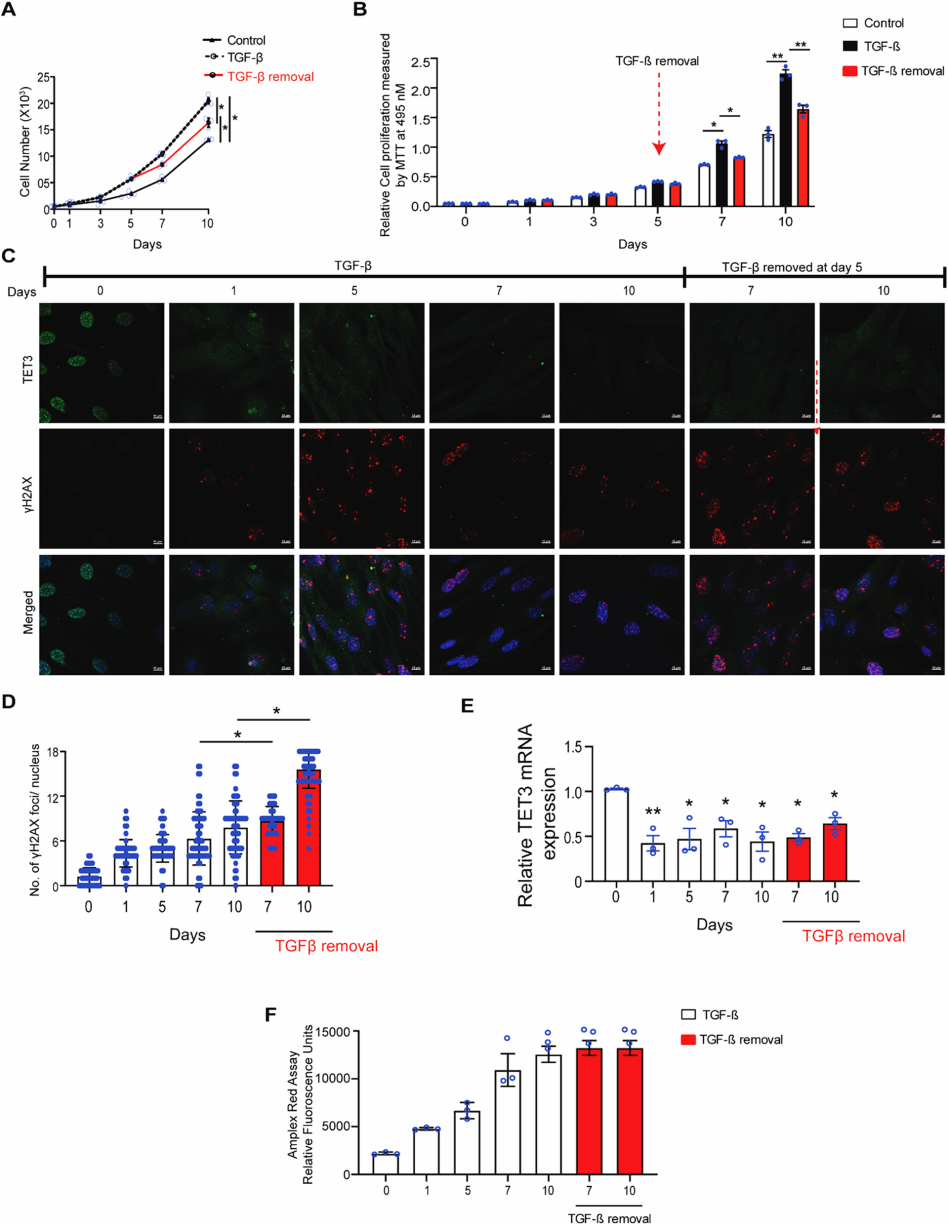
TGF-β affects cell proliferation, DNA damage, and TET3 expression in vitro in mCFs. **A** Representative graph shows the effect of TGF-β on cell number in mCFs from three biological experiments at indicative days. **B** Representative graph shows relative cell proliferation measured by MTT assay in control and TGF-β-treated mCFs from three biological experiments at indicative days. **C** Representative confocal images the effect of TGF-β on γH2AX and TET3 expression from three biological experiments in mCFs at indicative days. **D** Associated graph from Fig. 4e shows γH2AX foci in control and TGF-β-treated mCFs from three biological experiments at indicative days. **E** Relative TET3 mRNA expression upon TGF-β treatment from three biological experiments in mCFs at indicative days. **F** Fluorescence measurement using Amplex Red assay showing ROS intensity in TGF-β-treated mCFs from three biological experiments*.* All the represented experiments are done in triplicates, and statistical significance was calculated using one-way ANOVA. n.s. represents non-significant, and P values correspond to *p ≤ 0.05, **p ≤ 0.01, ***P ≤ 0.01

After establishing the role of TET3 in the HR-mediated DDR response in healthy cardiac fibroblasts, we proceeded to penetrate its role in fibrotic fibroblasts. TGF-β is a highly pleiotropic cytokine that is widely known to contribute to developing cardiac fibrosis [35]. Therefore, we mimicked a pro-fibrotic niche in the mCFs by treating them with 15 ng/mL of TGF-β every 48 h. Our results showed healthy mCFs upon pre-treatment with TGF-β, caused a rapid increase in both cell number and cell proliferation (Fig. 4A–B). Upon removal of the TGF-β on day 5, however, we observed a decrease in cell number and proliferation. Meanwhile, we noticed that exposure to TGF-β for 5 days results in the formation of DNA DSBs (Fig. 4C–D), which enrich upon removal of the TGF-β from the media on day 5 (Fig. 4D). These results suggest that TGF-β plays a role in the DDR response. Additionally, we noticed that failure to resolve the DNA damage upon TGF-β retraction on day 5 correlated with decreased cell number and decreased proliferation (Fig. 4A–B).

Our in vivo data demonstrated that fibrotic fibroblasts exhibit decreased TET3 expression (Fig. 1C). Coherent with this result, we next attempted to determine whether TGF-β treatment mimicking a fibrotic niche would result in decreased TET3 expression in the mCFs. Indeed, from our TET3 fluorescence staining and mRNA expression analysis, we confirmed downregulation of TET3 in mCFs upon TGF-β treatment (Fig. 4C and E). Interestingly, our results revealed that upon removal of TGF-β on day 5, expression of TET3 remained downregulated, suggesting an epigenetic mode of regulation (Fig. 4E).

Our study demonstrates that TGF-β exposure over a 5-day period conditions cardiac fibroblasts to enhance DNA repair mechanisms in response to oxidative stress, as evidenced by the Amplex Red assay (Fig. 4F). Throughout this period, we observed a continuous process of DSB resolution and accumulation, which can be attributed to chronic ROS buildup. This is supported by the gradual increase in DSBs on days 5 and 7. Upon withdrawal of TGF-β, there is a notable downregulation of the classical non-homologous end joining (c-NHEJ) repair efficacy (Fig. s4E). This downregulation leads to an increase in unresolved DNA damage and DSB accumulation. The withdrawal of TGF-β appears to disrupt the previously enhanced DNA repair mechanisms, resulting in a heightened level of DNA damage that the cells cannot efficiently repair. These findings highlight the dual role of TGF-β in modulating DNA repair mechanisms and emphasize the complex interplay between oxidative stress, ROS buildup, and DNA damage repair pathways in cardiac fibroblasts.

### TGF-β treatment results in decreased DSBs in mCFs

Our previous results demonstrated that TGF-ß treatment leads to decreased DNA DSBs in mCFs. Thus, we next hypothesized that the observed decrease in DSBs in mCFs upon TGF-ß treatment could be due to dampened DNA damage signaling or accelerated DSBs repair. To test this hypothesis, we challenged both TET3 knockdown mCFs and healthy mCFs with NCS either in the presence or absence of TGF-ß and followed the γH2AX kinetics. Our results demonstrated that short-term (up to 12 h) pre-treatment with TGF-β leads to a decreased accumulation of DNA DSBs in both control and TET3 knockdown mCFs (Fig. s4A–D). Additionally, our results indicated that the protective effect of TGF-ß persists in mCFs even when challenged with NCS, which can be visualized after 1 h (Fig. s4A–D), suggesting the involvement of TGF-ß in DNA repair pathways.

### TGF-β treatment results in shifts in DNA repair from HR to NHEJ in mCFs

**Fig. 5:**
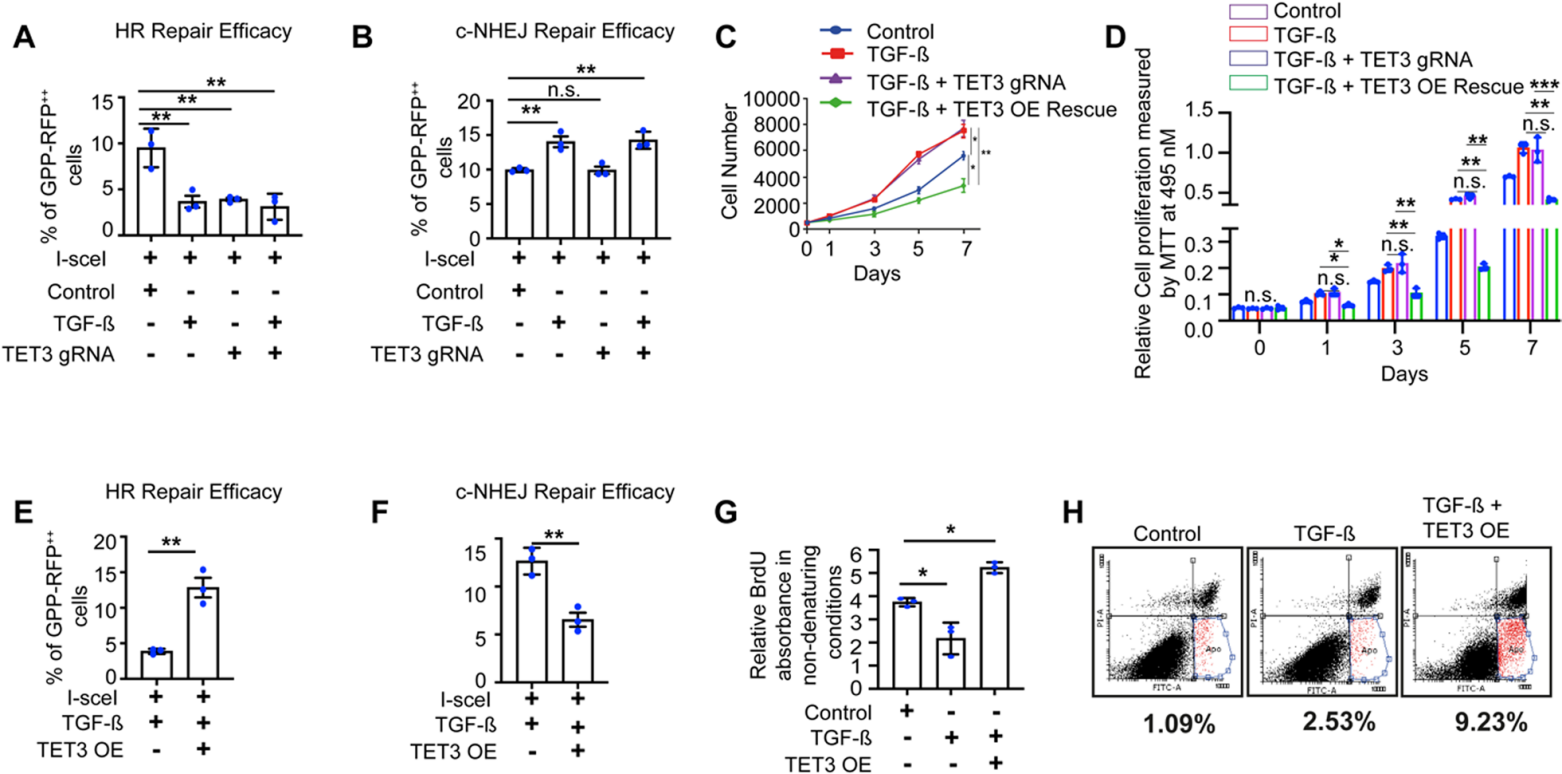
TGF-β increases NHEJ-mediated DNA repair facilitating proliferation in mCFs which can be rescued upon TET3 overexpression. **A** mCFs integrated with a DR-GFP HR reporter substrate were transfected in indicated conditions in combination with ISce-1, and analyzed for change in HR efficiency by scoring % of GFP/RFP double-positive cells using flow cytometry. **B** mCFs integrated with a pLCN-DSB NHEJ reporter substrate were transfected in indicated conditions in combination with ISce-1 and analyzed for change in NHEJ efficiency by scoring % of GFP/RFP double-positive cells using flow cytometry. **C** Representative graph shows the effect of TGF-β on cell number in control, TET3 knockdown and overexpressed mCFs over 7 days. **D** Representative graph shows the effect on cell proliferation measured by MTT assay in mCFs pre-treated with TGF-β in control, TET3 knockdown and overexpressed cells. **E** and **F** DNA repair constructs showing HR and NHEJ repair efficiency in mCFs pre-treated with in control, TET3 knockdown and TET3 overexpression conditions. **G** Graph represents BrdU incorporation in non-denaturing conditions, a direct readout for DNA end resection pre-treated with TGF-β in control, and TET3 overexpressed mCFs cells. **H** Annexin V assay showing change in apoptosis in TGF-β-treated mCFs with or without TET3 overexpression. Statistical significance was calculated using one-way ANOVA. n.s. represents non-significant and P values correspond to *p ≤ 0. 05, **p ≤ 0. 01

Studies in various cancer models have demonstrated that TGF-β can accelerate the clearance of DSBs via increased NHEJ repair [52]. Therefore, we hypothesized that the decreased DSBs observed in the mCFs upon TGF-β treatment could be attributable to accelerated DNA repair. To investigate this idea, we analyzed the HR and NHEJ DNA repair efficacies in the presence or absence of TGF-β in control and TET3 knockdown mCFs. Our results demonstrated that pre-treatment of TGF-β over 24 h results in a marked decrease in HR efficiency in mCFs (Fig. 5A). This result is in line with a previously published study of CD44** + **/CD24 cancer cells, in which a similar effect of TGF-β was reported [53].

Our findings showed that when TGF-β is treated in TET3 knockdown mCFs, there is a consistent reduction in the efficiency of homologous recombination (HR) as shown in Fig. 5A. However, TGF-β exposure in TET3 knockdown mCFs did not further reduce HR efficiency. This lack of additional decrease is likely because TGF-β itself reduces TET3 expression, as shown in Fig. 4E.

Next, we investigated the NHEJ repair efficacy in TGF-ß-treated mCFs and demonstrated that pre-treatment with TGF-β does indeed upregulate NHEJ repair efficacy (Fig. s4E). Furthermore, we noticed that this increase in NHEJ repair was maintained in TET3 knockdown cells treated in combination with TGF-β (Fig. 5B). Collectively from these results, we concluded that the decrease in DSBs observed in the mCFs upon TGF-β treatment occurred because of enhanced NHEJ repair efficiency and was not due to dampened DSBs sensing.

### TGF- β treatment promotes proliferation in fibroblasts, which can be rescued upon TET3 overexpression

Proliferation of cardiac fibroblasts is a necessity during cardiac fibrosis. Our previous results, however, demonstrated that knockdown of TET3 in the absence of fibrotic stimuli results in a decrease in cell number and a slower proliferation rate. Thus, we proceeded to investigate how TET3-deficient fibroblasts proliferate in a fibrotic state. For this purpose, we used TGF-β to mimic a fibrotic environment and compared the change in cell number and proliferation over 7 days in TET3 knockdown fibroblasts in the presence of TGF-β with the change in cell number and proliferation over 7 days in TET3 knockdown fibroblasts in the absence of TGF-β. Our cell counting and proliferation results indicated that TET3 knockdown mCFs treated with TGF-β have a higher cell number and a faster rate of proliferation than TET3 knockdown mCFs (Fig. 5C–D). Our results indicated that TET3 knockdown mCFs treated with TGF-β exhibit an unchanged cell number and, moreover, proliferate at the same rate as TGF-β-treated cells (Fig. 5C–D). As TGF-β also downregulates TET3 expression, our results further confirm that the loss of TET3 has no additive effect on the proliferation of mCFs during fibrosis. Thus, to evaluate the role of TET3 in proliferation during fibrosis, we next planned to overexpress TET3 in TGF-β-treated mCFs. Our results demonstrate that re-expression of TET3 in fibrotic conditions restrains both cell number and proliferation (Fig. 5C–D).

### TET3 overexpression in TGF-β-treated mCFs results in increased HR

Overexpression of TET3 in TGF-β-treated mCFs induces a reduction in cell number and cell proliferation. As TET3 is involved in the HR-mediated DNA repair response, we hypothesized that the observed reduction in cell number and cell proliferation could be associated with increased HR. Proper engagement of HR requires activation of G2M cell cycle checkpoint arrest [15]; therefore, we continued accessing the G2M phase and the H3s10p status of the mCFs. Our results demonstrated that, upon induction of DSBs, the control mCFs activated the proper G2/M arrest marked by decreased H3s10p activation (Fig. s5A–D), p-CHK2 foci (Fig. s5E–F) and increase in G2M population (Fig. s5G). Interestingly, we observed an increase in H3s10p in the TGF-β-treated mCFs as compared to the NCS-treated mCFs (Fig. s5A–D). Notably, upon overexpression of TET3 in the TGF-β-treated mCFs, we noticed a decrease in H3s10p, an increase in p-CHK2 foci (Fig. s5E–F), and a dramatic increase in the G2M population of cells (Fig. s5G), suggesting that the mCFs engaged in a shift from fast NHEJ-mediated repair to slow HR-mediated repair to fix the DSBs (Fig. s5D–E). To clarify whether the observed increase in G2M population and decrease in H3s10p upon TET3 overexpression in TGF-β-treated mCFs was indeed a consequence of a shift in DNA repair mechanisms, we again exploited HR and NHEJ repair reporter constructs. Our FACS results demonstrated an increase in HR efficacy upon TET3 overexpression in TGF-β-treated mCFs; unexpectedly, we also noticed a decrease in NHEJ efficacy (Fig. 5E–F). Recent reports suggest that an increase in HR-repair-mediated DNA end resection can be toxic to the survival of cells [54] and hence likely provides negative feedback not only to the cell proliferation but also to the engagement of the NHEJ repair machinery [55]. Our results are in sync with these observations, and we confirmed an increase in apoptosis upon TET3 overexpression in the TGF-β-treated mCFs (Fig. 5H), which was likely a consequence of increased toxic DNA end resection (Fig. 5G).

### Human fibrotic hearts lose TET3 expression

**Fig. 6:**
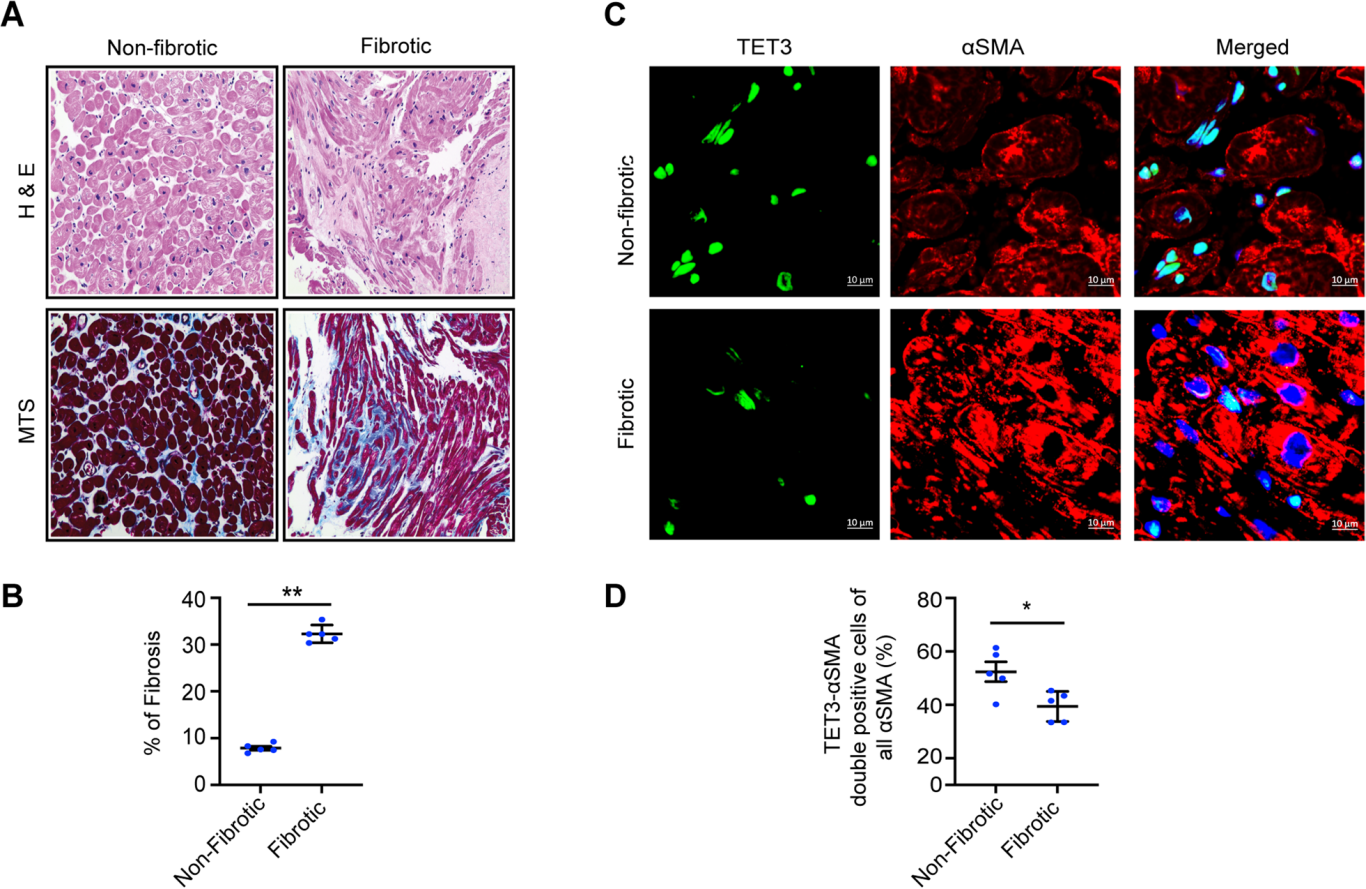
Human fibrotic fibroblasts have decreased TET3 expression. **A–B** MTS and H/E staining and associated graph representing % of the fibrotic area in non-fibrotic and fibrotic human hearts. **C** and **D** Confocal representative images and respective graph shows double staining of TET3 and αSMA in non-fibrotic and fibrotic human hearts. The arrow marks represent the fibroblasts positive or negative for TET3. Summarized quantitative findings are shown as mean ± SEM from 5 non-fibrotic, and 5 fibrotic human hearts. Each dot represents fibrotic area quantified from five independent field from each sample. Statistical significance was calculated using nonparametric two-tailed Student’s t test, and p values correspond to *p ≤ 0. 05. Scale bars represent 10 μm

To strengthen the clinical significance of the proposed study, we also examined the expression pattern of TET3 in the fibroblasts of non-fibrotic and fibrotic hearts of human patients. Using MTS staining, we confirmed and quantified the fibrosis in the human hearts. Using hematoxylin/eosin (H/E) staining, we further established the change in the morphology of the fibrotic human hearts (Fig. 6A–B). Using co-immunofluorescent staining with αSMA, we confirmed the expression of TET3 in the fibroblasts. Indeed, our results demonstrated that TET3 expression is downregulated in fibrotic human hearts (Fig. 6C–D), suggesting that the expression of TET3 is important for the proper functioning of a healthy human heart.

### Ischemic human hearts show decreased TET3 expression, which is negatively associated with RAD51 expression and positively associated with 53BP1 expression

Our in vivo data demonstrated downregulation of TET3 in fibrotic mouse hearts. Hence, our next aim was to evaluate the clinical significance of TET3 in healthy and diseased human hearts. Therefore, we examined the expression profile of TET3 using a publicly available microarray **(GSE57345)** dataset consisting of gene expression profiles collected from healthy and diseased human hearts [56]. The RNA samples analyzed in the microarray were obtained from the Myocardial Applied Genomics Network (MAGNet) consortium (www.med.upenn.edu/magnet/) and comprised information on TET3 expression in 135 ischemic left ventricles and 96 non-failing left ventricles. Consistent with our in vivo results in mouse hearts, we also uncovered a significant decrease in TET3 expression (*P* = 0.00000142) in human patient cohorts consisting of 135 ischemic left ventricles and 96 non-failing left ventricles (Fig. s6A). An increase in fibrosis is associated with increased COL4A1 and COL4A2 production. Our results revealed a negative correlation between the expression of TET3 and COL4A1 production, but not between the expression of TET1 or TET2 and COL4A1 production (Fig. s6B–D), suggesting that increased collagen production is associated with decreased TET3 expression. Additionally, we observed a positive correlation between the expression of TET3 and the expression of RAD51 (*P* = 0.0004; a marker for HR) and a negative correlation between the expression of TET3 and the expression of 53BP1 (*P* = 0.0005; a marker for NHEJ) in the same patient cohort (Fig. s6E–F), supporting our in vitro results in the mCFs, wherein loss of TET3 was associated with decreased HR efficacy but unchanged NHEJ efficacy.

## Discussion

Activation and proliferation of cardiac fibroblasts are the prime mediators of cardiac fibrosis [35]. Existing studies indicate that ROS and inflammatory cytokines produced during fibrogenesis not only result in increased proliferative stimuli but also contribute to DNA damage in the form of DSBs [7]. The presence of DSBs normally halts the cell cycle and activates DNA repair machinery to resolve the damage [15]; however, in pathological conditions, such as fibrosis, there is a continuous need for the proliferation of cardiac fibroblasts [35]. Hence, to sustain continuous cell proliferation, it is imperative to activate a specific DNA repair mechanism. In this study, we have unveiled that TET3 plays a pivotal role in modulating DNA damage response pathways, specifically by regulating homologous recombination (HR)-mediated DNA damage repair. Thus, while TGFß-1 shifted DNA damage response (DDR, which usually activates check point arrest) from homologous recombination (which requires check point arrest) to non-homologous end joining (NHEJ, which does not require check point arrest), TET3 counteracted this by shifting DDR back from NHEJ to HR. Consequently, the TGFß-1-induced switch to NHEJ-based DNA damage repair, accompanied with the loss of TET3, accelerates the removal of DNA damage and facilitates cell proliferation. Importantly, this increase in cell proliferation can be halted by re-expression of TET3.

TETs proteins are emerging as new sentinels of genomic stability [24, 57]. Notably, a recent study demonstrate that in mouse embryonic fibroblasts; of all the TET isoforms, TET3 plays a pivotal role in the efficient repair of camptothecin-induced DNA lesions, promoting genome stability [26]. Camptothecin is potent inducers of DNA SSBs or replication-stress-induced DNA breaks; hence, it is not completely known whether TET3 functions in DSBs repair or if it is recruited at the DSBs. In the present study, we demonstrate that TET3 recruits to DSBs. Additionally, using the readouts from the DNA repair reporter constructs, we established that TET3 facilitates HR-mediated but not NHEJ-mediated DNA repair. To our knowledge, in the context of cardiac fibroblasts, we are the first to report and demonstrate that TET3 recruits to DSBs and functions in HR-mediated DNA repair. Meanwhile, new studies highlighting a non-catalytic role of TET3 are emerging [39]. In our present study, we demonstrated that the loss of TET3 does not result in decreased 5-hmC levels even when challenged with exogenous DSBs, further confirming the non-catalytic role of TET3 in DNA repair. Results from a recent study in HEK293 and MEFs show a hydroxymethylation-independent role of TET1 in NHEJ-mediated DNA repair by forming a chromatin-associated complex with sin3a and hMOF transcriptional factors, and these results are coherent with our findings that catalytic-independent role of TET proteins can facilitate DNA repair [58]. Altogether, these studies suggest that both the catalytic-independent and the catalytic-dependent role of TET proteins are involved in DNA repair, but it may be highly plausible that the catalytic-dependent role is mostly restricted to the SSB or replication-stress-mediated DNA repair, whereas the catalytic-independent role is predominantly involved in repairing DSBs. TET3 demethylates 5-methylcytosine residues, particularly at gene promoters, creating a more open and accessible chromatin landscape [59]. This epigenetic modification enhances the binding of transcription factors and RNA polymerase II, promoting active transcription, and may facilitate the recruitment of HR repair machinery to these regions [60, 61]. Consequently, TET3 may play a crucial role in demethylating promoter regions, which is essential for initiating transcription-coupled homologous recombination (TC-HR) by maintaining an open chromatin state that supports both transcription and DNA repair [61, 62]. Therefore, TET3’s additional involvement in TC-HR cannot be ruled out; however, in this study we focus primarily on HR. HR repairs double-strand breaks throughout the genome using a homologous sequence as a template, whereas TC-HR specifically repairs breaks within actively transcribed genes by leveraging the transcription machinery to enhance repair efficiency [62, 63].While HR and TC-HR share core repair mechanisms, the repair factors involved are quite distinct and their regulation and activation are distinctly influenced by transcriptional activity and epigenetic modifications. Emerging reports show that TET2-mediated hydroxymethylation plays a crucial role in the maintenance of replicating fork origins [64]. TET proteins are highly redundant in their catalytic function, so its plausible TET3 might function in replication stress and the maintenance of replicating fork origins. One of the key findings of our present study was that TET3 relaxes chromatin. This finding is in line with a recent study, wherein the loss of TET3 is reported to facilitate heterochromatin formation [65]. Unrelaxed chromatin can hinder the proper operation of the DNA damage and repair response [51–66]. Our results are in sync, demonstrating impaired recruitment of RAD51 to DSBs in TET3 knockdown mCFs. In a relaxed chromatin architecture, the eviction of nucleosomes around the DSBs facilitates DNA end resection followed by strand invasion [67]. Our results are coherent with previously published findings, as we also observed that TET3-deficient cells with a compacted chromatin architecture exhibit inefficient DNA end resection; however, it is unclear whether compacted chromatin in TET3-deficient cells is attributable to physiological tight nucleosome sliding or to increased tail bridging of histones. Future investigation is warranted in this area.

In a physiological state, cardiac fibroblasts are more or less quiescent, but in pathological conditions, their proliferation is critical for fibrotic scarring. Interestingly, we observed that fibrotic fibroblasts exhibit an increased proliferation rate despite accumulating DNA damage. This led to the following question: How do fibrotic fibroblasts enter proliferation? To provide an answer to this question, we contemplated two hypotheses: first, they may be adapting and tolerating DNA damage, or, second, they may in fact be harnessing the fibrotic niche to accelerate DNA repair. Adaptation to DNA damage engenders genomic stability (GS), but, to our knowledge, there are no reports of GS during cardiac fibrosis. Moreover, we also looked for abnormal anaphasic bridges (an indicator of GI) in murine fibrotic hearts, and we detected no such abnormality, at least not in our sample. Therefore, we focused on the second hypothesis: that the fibrotic fibroblasts enter proliferation in a fibrotic niche due to a shift in DDR. To test this hypothesis, we treated cardiac fibroblasts in vitro with TGF-β (a key molecule driving fibrotic progression). Our data indicated that mCFs treated with short-term TGF-β tend to accelerate the clearance of DSBs by engaging in increased NHEJ-mediated DDR response, while long-term exposure leads to accumulation of DSBs. This observation, which is counterintuitive at first sight, is likely due to the fact that short-exposure to TGFß-1 does not induce DSBs (Fig. S4), but leads to activation of NHEJ which clears DSBs fast (Fig. 5). When cells are exposed to TGFß-1 long term, this causes DSBs (likely due to accumulation of ROS, Fig. 4), and even though NHEJ continues to be the predominant mechanism of DDR (Fig. 5), DSBs cannot be cleared up in a timely manner.

Interestingly, our results also demonstrated that mCFs display decreased TET3 expression upon long-term treatment with TGF-β. This is in line with previously published studies, wherein a similar effect of TGF-β has been observed [20, 21, 68–70]. So far, the exact mechanism that impairs TET3 expression even after withdrawal of TGFß-1 has not been addressed in the context of cardiac fibroblasts. One possible explanation is DNA methylation of the TET3 promoter, which has both SMAD binding sites and CpG islands. It would therefore be interesting to address whether these axes, either together or independently, regulate TET3 expression in mCFs. Altogether, these results led us to hypothesize that decreased TET3 expression is a strategy employed by the fibrotic fibroblasts to evade activation of the checkpoint-arrest-assisted HR-mediated repair response to promote increased cell proliferation; however, we also demonstrated that further knockdown of TET3 has no additive effect on the change in the cell proliferation state in TGF-β-treated mCFs. Interestingly, our results demonstrated that TET3 overexpression in TGF-β-treated mCFs also leads to a shift in NHEJ repair efficacy. We propose that this result could be a consequence of increased DNA end resection in relaxed chromatin. This idea is consistent with a recent study, wherein knockdown of CCRA2 was found to decrease NHEJ repair efficacy due to an increase in HR-mediated DNA end resection [55]. In summary, based on the findings derived from our current study, we can conclude that TET3 serves as a limiting factor for cell proliferation in fibroblasts by promoting HR-mediated DNA repair.

## Conclusion

The study highlights the critical role of TET3 in modulating DNA damage response mechanisms in cardiac fibrosis. By shifting the DDR from c-NHEJ to an HR pathway, TET3 overexpression in a fibrotic niche reduces the excessive proliferation of cardiac fibroblasts, which is a hallmark of fibrosis. These findings not only underscore the protective epigenetic role of TET3 in organ fibrosis but also suggest potential therapeutic strategies targeting TET3 to mitigate the adverse effects of cardiac fibrosis.

### Limitation of the study

The sensitivity of the assay used to measure total 5-hydroxymethylcytosine (5-hmC) levels is crucial due to the inherently low levels of hydroxymethylation. Although we utilized a hydroxymethylation-specific kit, typically based on ELISA analysis, the variability in sensitivity may limit the detection of small decreases in 5-hmC levels, especially below 0.05%. Additionally, while the study focuses on TET3’s role in DNA repair in mCFs, the potential involvement of its canonical hydroxymethylation function cannot be entirely dismissed, necessitating a more detailed study in this respect.

### Supplementary Information

Additional file 1.

Additional file 2.

Additional file 3.

Additional file 4.

Additional file 5.

Additional file 6.

Additional file 7.

## Data Availability

The datasets used and/or analyzed during the current study are available from the corresponding author on reasonable request.
