# Supplementary figures and images for: Loss of tet methyl cytosine dioxygenase 3 (TET3) enhances cardiac fibrosis via modulating the DNA damage repair response
Source: Clin Epigenetics. 2024 Aug 27;16:119. doi: 10.1186/s13148-024-01719-6 (PMC11350970; doi:10.1186/s13148-024-01719-6)

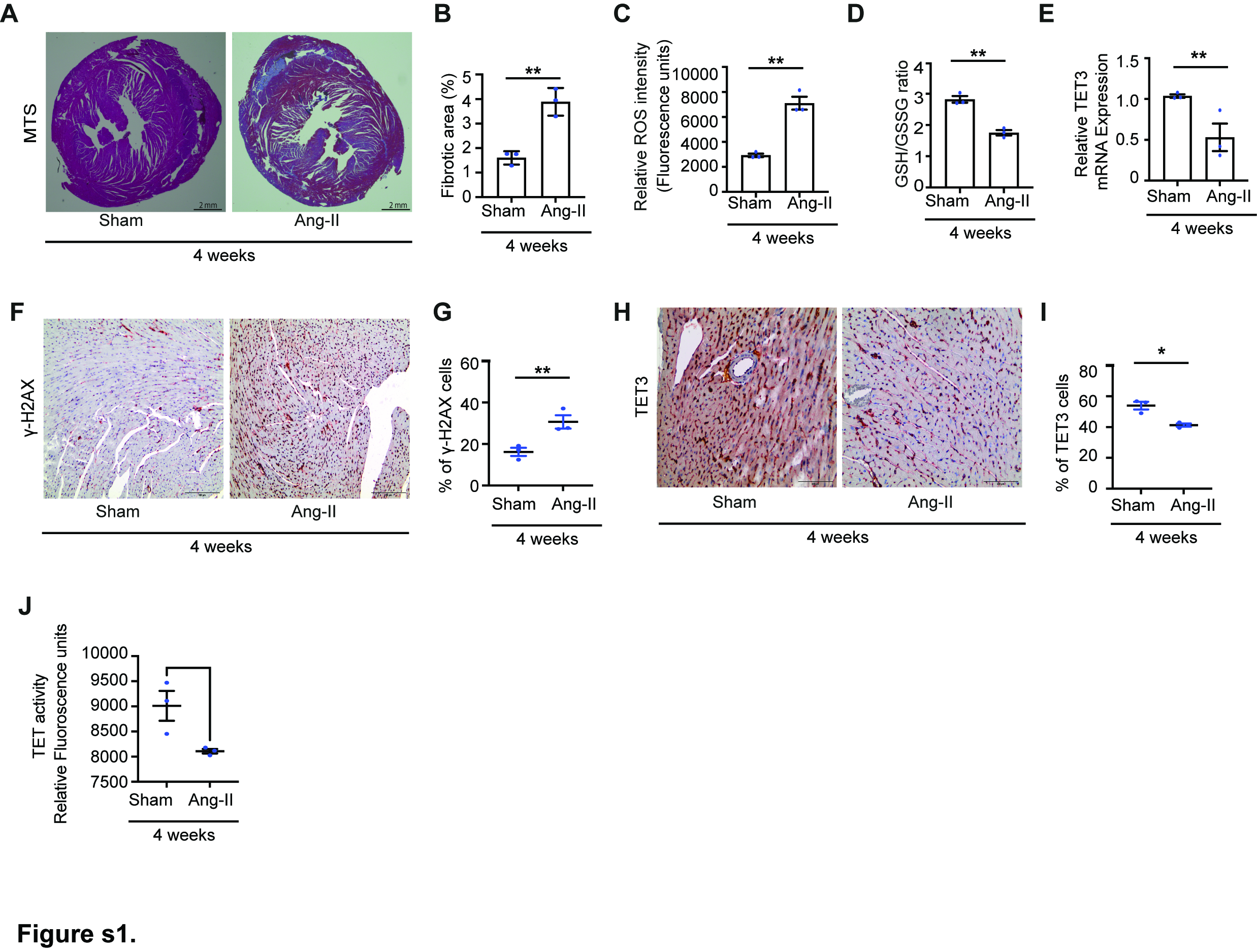

Supplement: Supplementary file 1 — Additional file 1. [file 13148_2024_1719_MOESM1_ESM.tif]

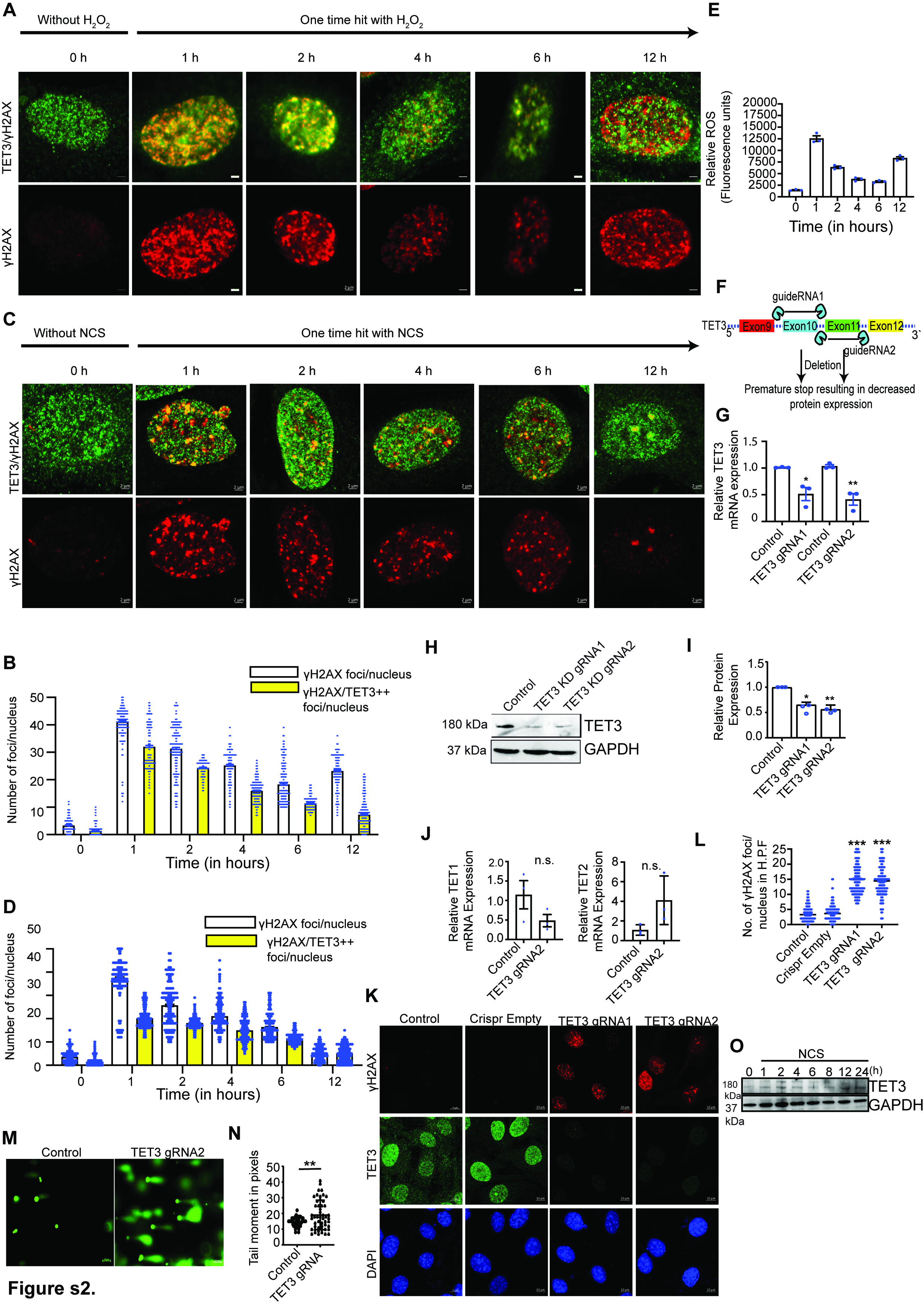

Supplement: Supplementary file 2 — Additional file 2. [file 13148_2024_1719_MOESM2_ESM.tif]

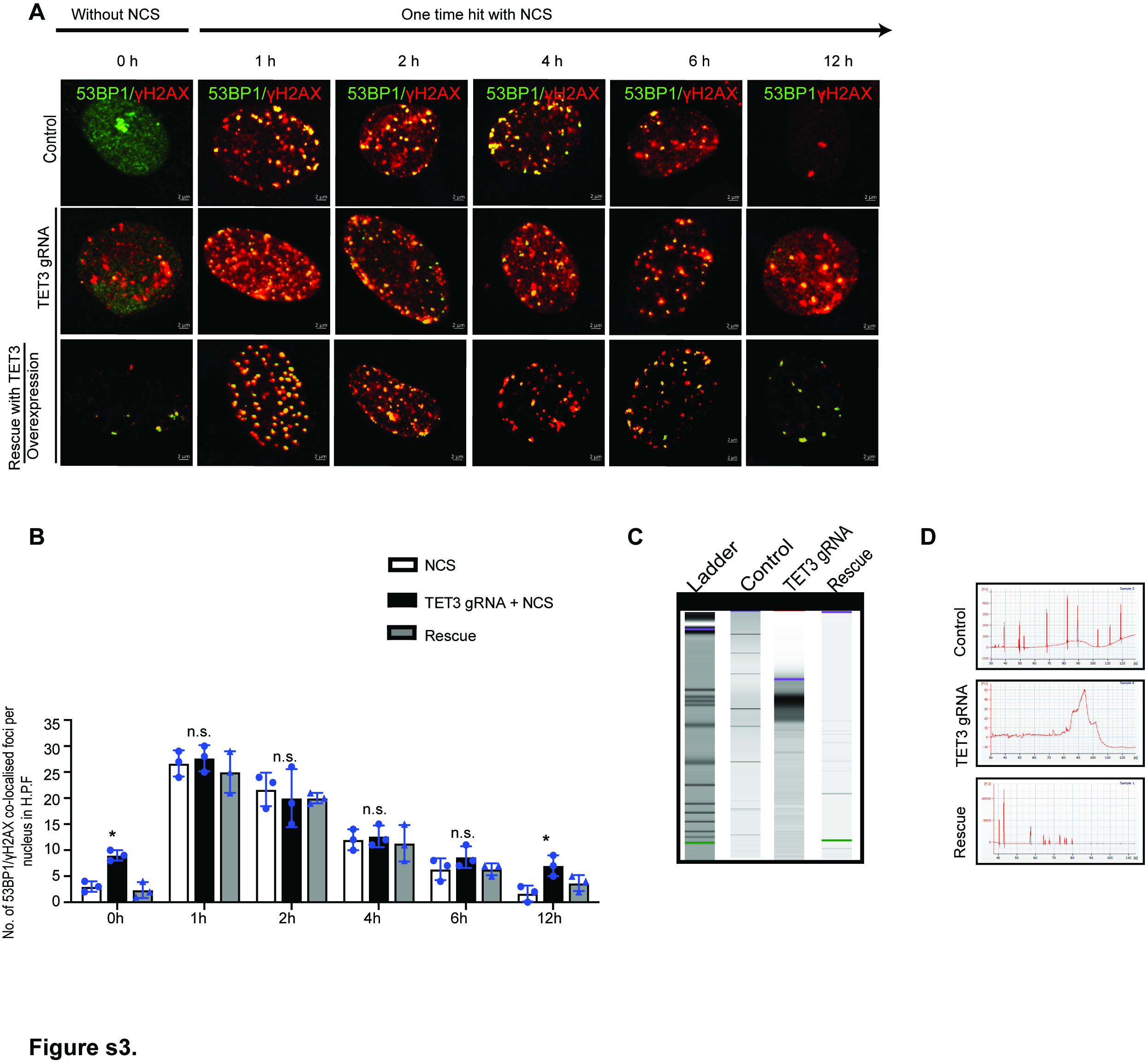

Supplement: Supplementary file 3 — Additional file 3. [file 13148_2024_1719_MOESM3_ESM.tif]

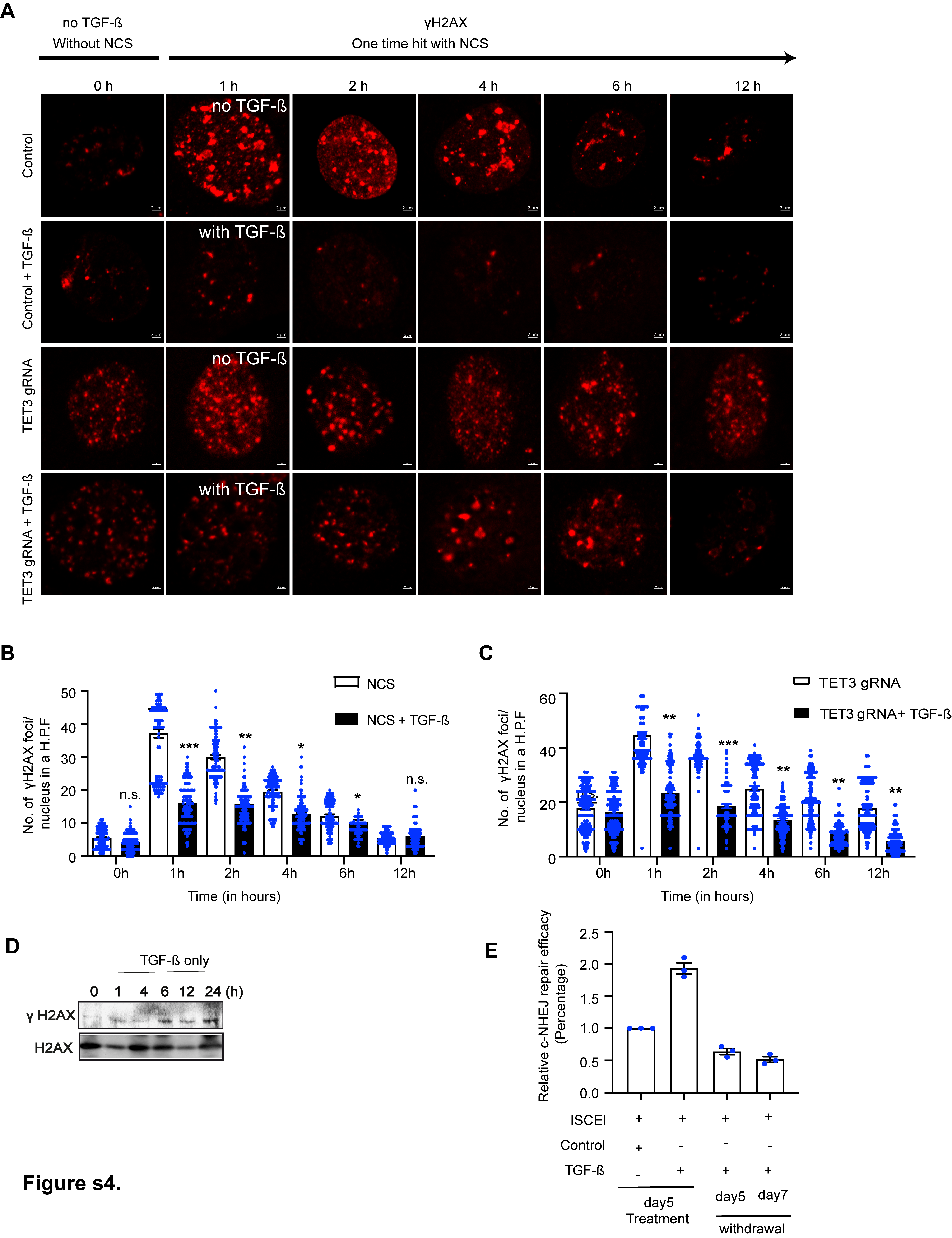

Supplement: Supplementary file 4 — Additional file 4. [file 13148_2024_1719_MOESM4_ESM.tif]

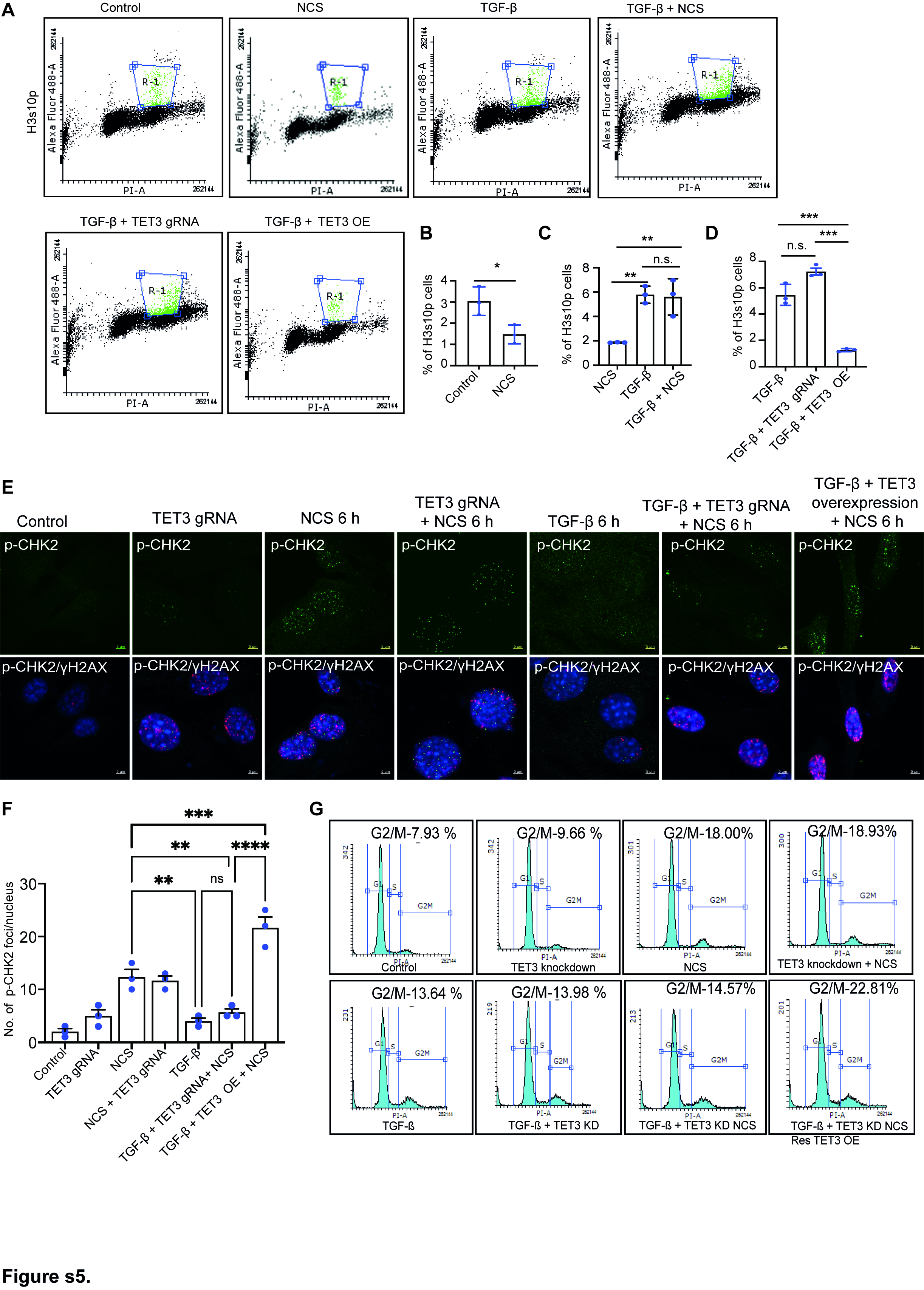

Supplement: Supplementary file 5 — Additional file 5. [file 13148_2024_1719_MOESM5_ESM.tif]

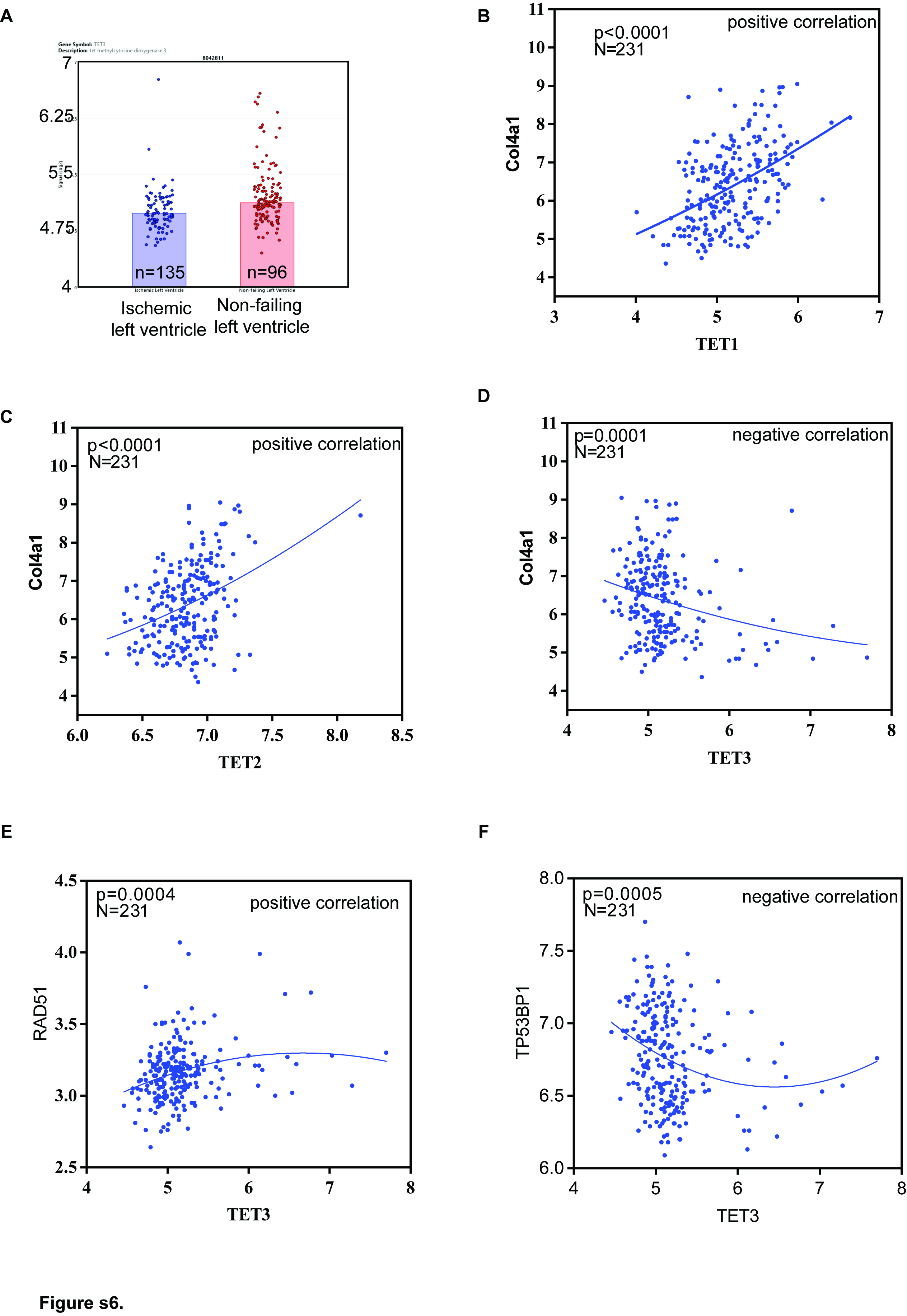

Supplement: Supplementary file 6 — Additional file 6. [file 13148_2024_1719_MOESM6_ESM.tif]
